# Characterization of the spore surface and exosporium proteins of *Clostridium sporogenes*; implications for *Clostridium botulinum* group I strains

**DOI:** 10.1016/j.fm.2016.06.003

**Published:** 2016-10

**Authors:** Thamarai K. Janganan, Nic Mullin, Svetomir B. Tzokov, Sandra Stringer, Robert P. Fagan, Jamie K. Hobbs, Anne Moir, Per A. Bullough

**Affiliations:** a Krebs Institute, University of Sheffield, Sheffield S10 2TN, UK; b Dept. of Molecular Biology and Biotechnology, University of Sheffield, Sheffield S10 2TN, UK; c Dept. of Physics and Astronomy, University of Sheffield, Sheffield S3 7RH, UK; d Institute of Food Research, Norwich Research Park, Norwich NR4 7UA, UK

**Keywords:** Spore appendages, Two dimensional crystals, Cysteine rich proteins

## Abstract

*Clostridium sporogenes* is a non-pathogenic close relative and surrogate for Group I (proteolytic) neurotoxin-producing *Clostridium botulinum* strains. The exosporium, the sac-like outermost layer of spores of these species, is likely to contribute to adhesion, dissemination, and virulence. A paracrystalline array, hairy nap, and several appendages were detected in the exosporium of *C*. *sporogenes* strain NCIMB 701792 by EM and AFM. The protein composition of purified exosporium was explored by LC-MS/MS of tryptic peptides from major individual SDS-PAGE-separated protein bands, and from bulk exosporium. Two high molecular weight protein bands both contained the same protein with a collagen-like repeat domain, the probable constituent of the hairy nap, as well as cysteine-rich proteins CsxA and CsxB. A third cysteine-rich protein (CsxC) was also identified. These three proteins are also encoded in *C*. *botulinum* Prevot 594, and homologues (75–100% amino acid identity) are encoded in many other Group I strains. This work provides the first insight into the likely composition and organization of the exosporium of Group I *C*. *botulinum* spores.

## Highlights

• *Clostridium sporogenes* spores have a paracrystalline outer surface (exosporium).
• The spore surface is decorated with a variety of filaments and appendages.
• Appendages include long beaded filaments of unknown function.
• BclA-like exosporium proteins with collagen-like repeat domains are present.
• Novel cysteine-rich exosporium proteins may compose the exosporium basal array.

## 1 Introduction

Endospores are produced by *Bacillus* and *Clostridium* spp; their extreme resistance properties contribute to their persistence and dissemination in the environment. A cellular core is surrounded by a thick layer of peptidoglycan cortex, a proteinaceous spore coat, and in many cases a looser outermost layer, the exosporium.

The only exosporium layer studied in detail is that of *Bacillus anthracis* spores and the spores of the closely related *Bacillus cereus* and *Bacillus thuringiensis*, where it contains an outer hairy nap and a paracrystalline basal layer with hexagonal arrays (Stewart, 2015). The BclA glycoprotein of *B*. *anthracis*, which contains a collagen-like repeat (CLR) domain, is the major contributor to the hairy nap layer of the exosporium (Sylvestre et al., 2003). This protein is likely to be involved in adherence and entry into the host (Xue et al., 2011) and influences spore surface properties (Lequette et al., 2011). A second CLR-containing glycoprotein BclB is also present in *B*. *anthracis* exosporium (Thompson et al., 2012). The cysteine-rich ExsY protein is essential for the formation of exosporium (Boydston et al., 2006, Johnson et al., 2006).

There is little information on the exosporium of *Clostridia*, with the recent exception of *Clostridium difficile*. In that species a BclA-like protein, BclA1, is present and a cysteine-rich protein, CdeC, is required for morphogenesis of the coat and of an exosporium layer (Paredes-Sabja et al., 2014).

*Clostridium botulinum* is responsible for major food poisoning by producing lethal neurotoxin (Peck et al., 2011) and is classified as a potential bioterror agent. It is essential to understand the structure and composition of spore surface layers, to underpin development of detection and inactivation regimes. As yet, only limited information is available for *C*. *botulinum* exosporium. For example, the exosporium of proteolytic *C*. *botulinum* type A strain 190L demonstrates a hexagonal array (Masuda et al., 1980) and is resistant to urea, DTT, SDS and proteolytic enzymes (Takumi et al., 1979). For practical reasons, we have chosen to study *Clostridium sporogenes*, a non-pathogenic and widely used surrogate for Group I (proteolytic) *C*. *botulinum* (Peck et al., 2011). A recent phylogenetic analysis, using complete and unfinished whole genome sequences (Weigand et al., 2015), shows that within *C*. *botulinum* Group I, a major cluster of strains (including Hall, Langeland and Loch Maree) can be distinguished from the major *C*. *sporogenes* cluster, which itself does include some *C*. *botulinum* toxigenic strains, such as Prevot 1662, Prevot 594 (Smith et al., 2015), Osaka 05 and ATCC 51387. *C*. *sporogenes* NCIMB 701792 (NCDO 1792), the subject of this study, has been included in a microarray study of genome relatedness within *C*. *botulinum* Group I and *C*. *sporogenes* strains (Carter and Peck, 2015), and we have found the exosporium of this *C*. *sporogenes* strain amenable to proteomic and structural analysis.

## 2 Materials and methods

### 2.1 Strains, growth conditions and media

*C*. *sporogenes* NCIMB 701792 (NCDO 1792) was grown on BHIS (Brain heart infusion supplemented with 0.1% l-cysteine and 5 mg/ml yeast extract) agar as previously described in (Smith et al., 1981) and incubated at 37 °C overnight in an anaerobic chamber with 10% H_2_, 10% CO_2_ and 80% N_2_.

### 2.2 Spore preparation and harvest

A single colony from BHIS agar was inoculated into TGY (Tryptose glucose yeast extract) broth. After overnight growth at 37 °C, 1.5 ml was added to 15 ml of SMC (Sporulation medium) broth (Permpoonpattana et al., 2011), and grown to an OD_600_ of 0.4–0.7. Aliquots (0.1 ml) were spread on SMC agar, and incubated at 37 °C for 1 week. Spores from the agar surface were harvested by resuspension in 3 ml ice-cold sterile distilled water, and water-washed 10 times to remove vegetative cells and debris, then separated from remaining vegetative cells by gradient centrifugation in 20%–50% Histodenz™ (Sigma). The spores were washed twice as above with water to remove the Histodenz™. Preparations (>99% free spores) were stored in sterile distilled water at 4 °C.

### 2.3 Exosporium preparation

Spores were diluted in spore resuspension buffer (SRB) (50 mM Tris HCl pH-7.5, 500 mM NaCl, 0.5 mM EDTA, and 1 mM PMSF) to 80 ml at OD_600_ of 2–3, French pressed twice at 16,000 psi, and the suspension centrifuged at 10,000 xg for 15 min to pellet the spores. The supernatant was reserved, and pellets were washed twice more in SRB. All supernatants were pooled and concentrated to 3 ml using centrifugal concentrators (Sartorius, 10 kDa cutoff). Concentrated exosporium was diluted with 4 vol of 20% urografin R-370 (Schering), layered onto 50% urografin, and centrifuged at 16,000 xg for 30 min. The top yellow layer containing the exosporium was collected, dialysed against water, and centrifuged at 100,000 xg for 2 h; the pellet containing the purified exosporium fragments was resuspended in 0.5 ml SRB and stored at −20 °C.

### 2.4 Salt and SDS wash of the exosporium fragments

Exosporium fragments purified as described in Section 2.3 were washed sequentially in exosporium wash buffers, using high salt and then SDS to remove loosely associated proteins. These washes, modified from Todd et al. (2003), were 1: 50 mM Tris pH-7.5, 0.5 M KCl, 0.25 M NaCl, 1 mM EDTA, 1% glycerol and 1 mM PMSF; 2: 1 M NaCl; 3: 50 mM Tris pH-7.5, 10 mM EDTA, 0.1% SDS and 1 mM PMSF and 4: 50 mM Tris pH-7.5, 10 mM EDTA and 1 mM PMSF (to remove SDS). Each wash was centrifuged at 100,000 xg for 2 h. Washes 3 and 4 were repeated. The final pellet (washed exosporium) was resuspended in 500 μl of 50 mM Tris pH-7.5, 10 mM EDTA, and 1 mM PMSF, passed through a 21G needle several times and stored at −20 °C.

### 2.5 Sonication to release exosporium fragments

Spores diluted in SRB were sonicated for 20 cycles of 15 s with 15 s cooling and then centrifuged at 1000 xg for 10 min to pellet the spores. The supernatants containing exosporium fragments were used for EM studies.

### 2.6 Protein concentration determination

Protein concentration was measured using the BCA protein assay (Pierce).

### 2.7 Gel electrophoresis of exosporium proteins

Aliquots (15–53 μg) of washed exosporium were vacuum dried at 30 °C for 20 min, then resuspended in 15 μl of urea solubilisation buffer (25 mM Tris pH-8, 8 M urea, 500 mM NaCl, 4 M DTT & 10% SDS) and heated at 95 °C for 20 min. After addition of 5 μl of 4X LDS sample buffer (Invitrogen), the sample was loaded on a 4–12% gradient NuPAGE Bis-Tris pre-cast SDS gel (Invitrogen), with MOPS running buffer. Gels were stained with SYPRO Ruby, visualized under UV, then further stained with Coomassie blue R-250.

### 2.8 Identification of exosporium proteins

#### 2.8.1 Proteins in major bands from SDS-PAGE

Samples (1.5 mm diameter), excised from selected gel bands, were digested with trypsin following cysteine derivatisation; peptides were analysed by nano-liquid chromatography/mass spectrometry/mass spectrometry (LC-MS/MS). Protein identification was from a database containing 7995 *C*. *sporogenes* sequences downloaded from UniProtKB. The detailed protocol is described in SI-1.

#### 2.8.2 Protein extraction directly from bulk exosporium

Proteins solubilized from washed exosporium by incubation in 0.5% deoxycholic acid, 12 mM N-lauroylsarcosine and 50 mM ammonium bicarbonate were trypsin digested after cysteine derivatisation, subjected to nano-LC-MS/MS analysis, and protein identified as for the gel-bands. The detailed protocol is described in SI-2.

### 2.9 Electron microscopy (EM)

Diluted whole spores or purified exosporium (3 μl) were applied to glow discharged carbon-coated Cu/Pd grids; after 1 min the grid was blotted, washed once with (0.75%) uranyl formate and then stained for 20 s. Excess stain was removed by blotting followed by vacuum drying and the grid examined in a Philips CM100 electron microscope operating at 100 kV. Images were recorded on a Gatan MultiScan 794 1 k × 1 k CCD camera at between 3000 and 52,000× magnification and 500–1200 nm underfocus. 10 length measurements from hairy nap, and other filaments were taken and the mean values with standard deviations are shown in the data.

### 2.10 Atomic force microscopy (AFM)

A suspension of exosporium fragments (Section 2.4) was incubated on freshly cleaved mica (Agar Scientific) for 20 min, then washed with 10 × 1 ml of HPLC grade water (Sigma Aldrich). Samples imaged in water were used without further preparation; samples imaged in air were blown dry with filtered nitrogen. Imaging in water was performed using a Dimension FastScan AFM (Bruker) and FastScan D probes (nominal force constant and resonant frequency 0.25 N/m and 110 kHz in water, respectively) in tapping mode with a free amplitude of approximately 1.2 nm and a set point of 80–90% of this value. Imaging in air was performed using a Multimode (Bruker) or NanoWizard^®^ 3 (JPK) AFM in tapping mode using TESPA probes (Bruker) with a nominal force constant and resonant frequency of 40 N/m and 320 kHz, respectively. The free amplitude was approximately 8 nm and images were acquired with a set point 90–95%. In both environments, the feedback gains, scan rate, set point and Z range were adjusted for optimal image quality while scanning. Height and phase images were acquired simultaneously and processed (cropping, flattening, plane fitting) using NanoScope Analysis or JPK DP software.

## 3 Results and discussion

### 3.1 *C*. *sporogenes* spore surface features revealed by EM

**Fig. 1:**
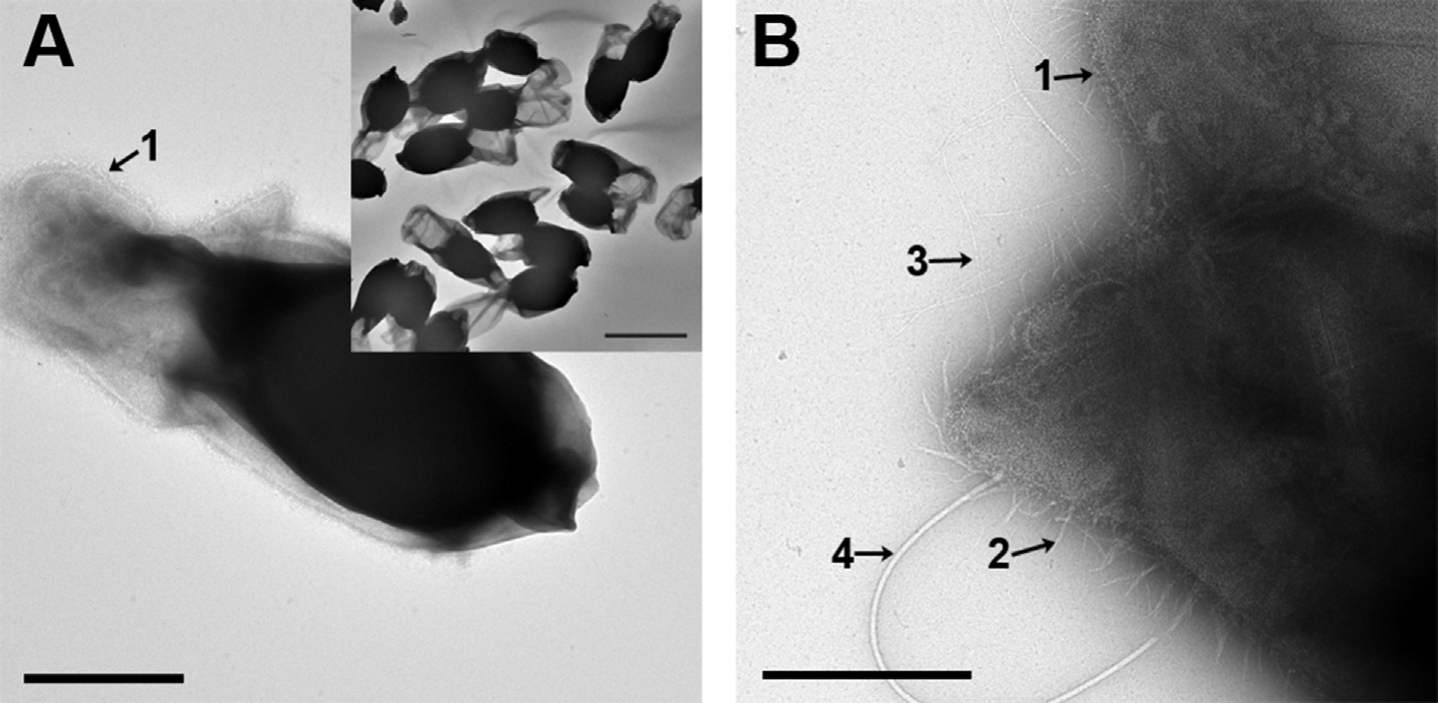
**Negative stain electron microscopy of whole spores and fragments of the exosporium of *C*. *sporogene*s NCIMB 701792**. A. A whole spore, showing the electron-dense core with the folded sac-like exosporium extended at one pole. Scale bar, 0.5 μm. A ‘hairy nap’ is present on the surface of the exosporium (arrow 1). Inset shows a lower magnification, wider view. Scale bar, 2 μm. B. High magnification image of part of a whole spore edge showing an area of exosporium with various surface features labeled: 1, hairy nap; 2, intermediate fibril; 3, beaded fibril; 4, large appendage. Scale bar, 0.5 μm.

**Fig. 2:**
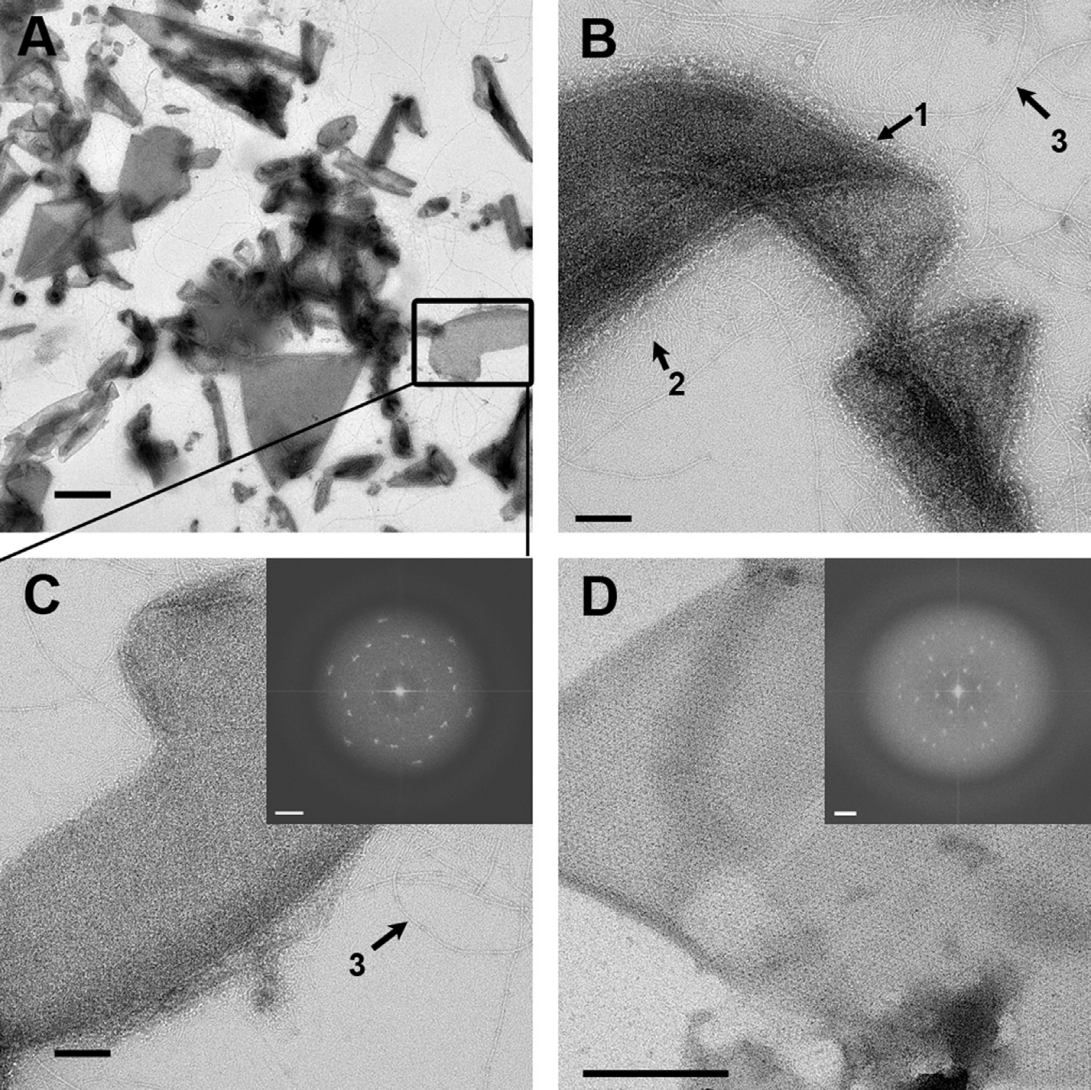
**Negative stain electron microscopy of purified fragments of exosporium**. A. Fragments of exosporium produced by French press, followed by a series of salt and SDS washes. The box indicates the fragment magnified in Fig. 2C. Scale bar, 0.5 μm. B. Hairy nap (1), intermediate fibrils (2) and beaded fibrils (3) are visible on the surface of an exosporium fragment. Scale bar, 100 nm. C. Magnified, boxed area from Fig. 2A; a hairy nap is visible all along the perimeter, suggesting that the entire surface is covered. It also shows an attached beaded fibril (3). Scale bar, 100 nm. Inset shows a Fourier transform from an area of the image. Scale bar, 0.104 nm^−1^. D. Fragment of unwashed exosporium produced by the sonication method. It reveals a well-contrasted and well-ordered hexagonal lattice. There is no indication of any hairy nap on this fragment. Scale bar, 200 nm. Inset shows a Fourier transform from an area of the image. Scale bar, 0.104 nm^−1^.

The spores of *C*. *sporogenes* (Fig. 1) are enveloped by an exosporium that is more extended at one pole. The exosporium has a ‘hairy nap’ that appears uniform (30 ± 5 nm deep) along the perimeter of the exosporium when viewed in projection, so we infer that it covers the entire surface (Fig. 1A). At higher magnification (Fig. 1B), other features of the exosporium surface become visible including the hair-like nap (arrow 1) and intermediate fibrils (arrow 2). In addition, beaded fibrils, which display a regular pattern of bead-like structures along their length (arrow 3; clearly visible in higher magnification in Fig. 2B and C), and a large appendage up to 1–2 μm long with a diameter of 20 nm (arrow 4) are present on the spore surface.

### 3.2 Surface features of exosporium fragments revealed by EM

**Fig. 3:**
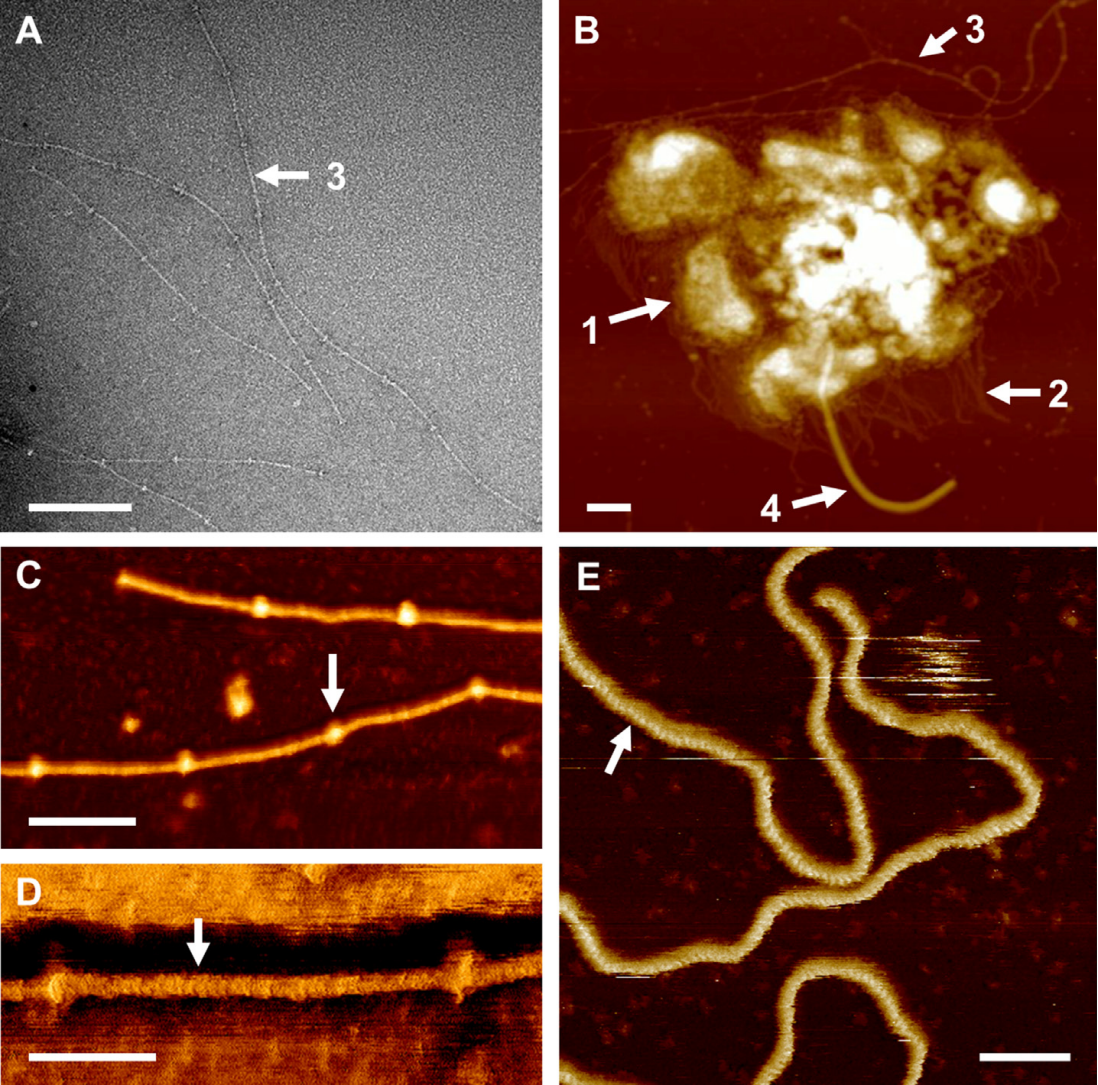
Surface decorations of the exosporium. A. A high magnification negative stain electron micrograph of washed exosporium shows a beaded fibril with regular bead-like repeats at approximately 70 nm intervals. Scale bar, 100 nm. B. AFM height image of a fragment of washed exosporium. Four different surface features are indicated by white arrows with numbering: 1, hairy nap; 2, intermediate fibril; 3, beaded fibril; 4, a large appendage. The image was recorded in tapping mode in air. Dark to bright variation corresponds to a height of 30 nm. Scale bar, 100 nm. C. AFM height image recorded as in (B) shows beaded fibril with the beads (arrow). The distance between two beads is approximately 70 nm; dark to bright variation in height is 4.5 nm. Scale bar 50 nm. D. Cropped region of the AFM phase image recorded simultaneously with the height image shown in C, shows internal repeats of approximately 2 nm in the beaded fibril (arrow). Dark to bright variation in phase is 10.8°. Scale bar 20 nm. E. AFM height image taken in tapping mode in water shows beaded fibrils with internal repeats of approximately 2 nm (arrow). The beads observed in tapping mode in air were not observed in water. Dark to bright variation in height is 4.36 nm. Scale bar 20 nm.

EM analysis of purified, washed exosporium confirmed the presence of highly enriched exosporium fragments (Fig. 2A). A higher magnification view is shown in Fig. 2B and C. The fragments tend to fold, revealing surface features clearly at the fold perimeter, including a hairy nap (arrow 1), intermediate fibrils approximately 80–200 nm long (arrow 2), and beaded fibrils up to 1–2 μm long (arrow 3, Fig. 2B). Most fragments showed an underlying ordered crystal lattice (insets, Fig. 2C and D). In a small proportion (10%) of fragments of sonicated exosporium, the lattice showed sufficient contrast that it could be clearly discerned by eye (Fig. 2D); in these cases the hairy nap was detached and there was less disordered background. Fig. 3A shows more detail of the beaded fibrils with ‘beads’ at regular intervals of 70 ± 2 nm (arrow 3). The diameter across the fibril and beaded area is 6 ± 1 nm and 11 ± 1 nm, respectively. It is unclear whether the beaded fibrils and large appendages emanate directly from the exosporium or from deeper within the spore. However, it is clear that they can remain associated with the exosporium when fragmented.

### 3.3 Surface features of exosporium fragments and appendages revealed by AFM

An AFM image of exosporium fragments in air shows a nap-like fringe, approximately 20–30 nm deep (Fig. 3B; arrow 1), on the spore surface. Other appendages include (i) intermediate fibrils ca. 80–200 nm in length (Fig. 3B; arrow 2) and (ii) beaded fibrils, punctuated with ‘bead-like’ elements (Fig. 3B, C and D; arrow 3) at an average interval of 68 ± 2 nm. There is a shorter periodic feature (repeat of 1.9 ± 0.2 nm) on the fibril, shown in Fig. 3D. The measured height and width of the beaded fibril in air is approximately 2 nm and 8 nm, respectively, and of the beaded area 3 nm and 14 nm, respectively (Fig. 3D). We also saw the approximate 2 nm repeat (2.0 ± 0.2 nm) when fibrils were imaged in water (Fig. 3E). However, we found no evidence of the regular ‘beads’. It is possible that the beads arise through some contractile mechanism upon dehydration, as they are also seen under negative stain EM. In addition, a large appendage, with height 6–9 nm, width 20–30 nm and up to 1–2 μm long, is seen on the surface of the exosporium (Fig. 3B; arrow 4). When measured with AFM the height and width of these appendages are significantly smaller and larger (respectively) than the diameter measured by EM. We attribute these effects to convolution of the fibril with the imaging tip; this has been shown to significantly increase the measured width and reduce the measured height when imaging features that are comparable in size, or smaller than, the length scale of the tip-surface interaction area. This effect has been shown to be particularly prevalent when imaging isolated structures on otherwise flat surfaces (Santos et al., 2011).

### 3.4 *C*. *sporogenes* exosporium protein profiles

**Fig. 4:**
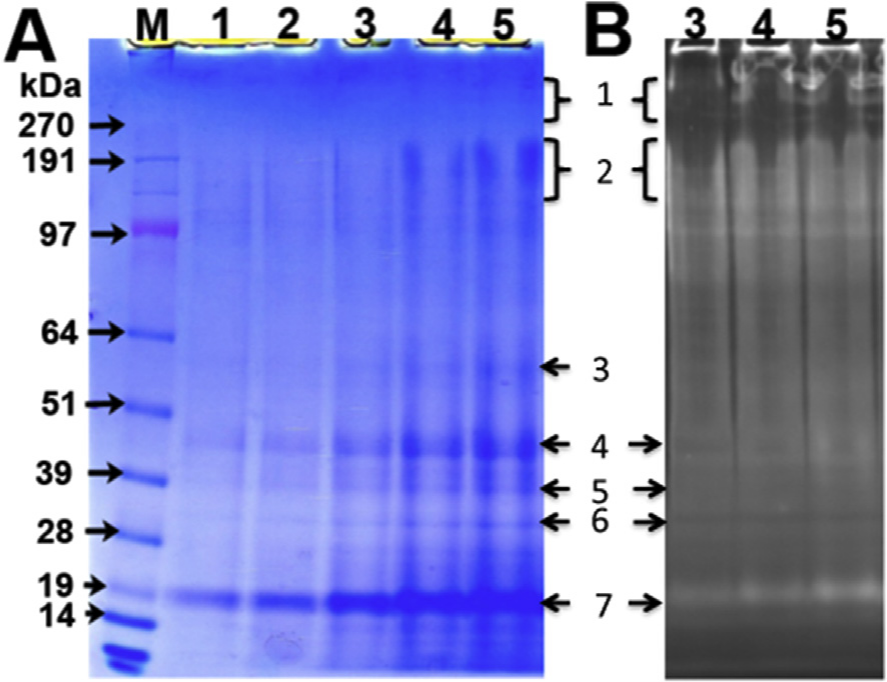
**Protein profile of salt and SDS washed exosporium**. A. Aliquots (15–53 μg) of washed exosporium were vacuum dried, resuspended in 15 μl of solubilisation buffer (25 mM Tris pH-8, 8 M urea, 500 mM NaCl, 4 M DTT, 10% SDS) and boiled at 95 °C for 20 min, loaded on 4–12% gradient (NuPAGE) gel and resolved with MOPS buffer. The gel was stained with Coomassie brilliant blue R-250. The marked bands (1–7) were excised and proteins identified by LC-MS/MS. Lane M, protein marker, lane 1,12.5 μg; lane 2, 25 μg; lane 3, 32.5 μg; lane 4, 45 μg and lane 5, 52.5 μg of exosporium protein loaded. B. The gel shown in Fig. 4A (lanes 3–5) was stained with SYPRO Ruby and visualized by ultraviolet light. Bands above 191 kDa are clearly visible (For interpretation of the references to colour in this figure legend, the reader is referred to the web version of this article.)

Exosporium fragments (Section 2.3) were dried and resuspended in a denaturing buffer containing 8 M urea, 4 M DTT and 10% SDS and heated (Section 2.7) before separation of proteins by SDS-PAGE (Fig. S1). Six of the most strongly stained bands were excised for further analysis. In order to remove loosely attached or contaminating proteins, these exosporium fragments were further subjected to high salt and SDS washes (Section 2.4), then resuspended and heated as above before separation on SDS-PAGE. The protein profile of washed exosporium fragments (Fig. 4A) contained bands ranging from <19 kDa to >190 kDa. The region above 190 kDa was poorly stained by Coomassie, but material at 200 to >300 kDa was clearly visible on the SYPRO Ruby stained gel (Fig. 4B). A significant proportion of the material remained in high molecular weight species, despite the harsh solubilisation conditions. The smeared regions (>200 kDa) at the top of the gel (band 1) and from 150 to 200 kDa (band 2) along with 5 smaller individual bands (bands 3–7) were excised for protein identification.

### 3.5 Identification of proteins from excised gel bands

Proteins were analysed by LC-MS/MS of trypsin digests (Section 2.8.1). As the genome sequence of strain *C*. *sporogenes* NCIMB 701792 is not available, the identifications were made using proteins of *C*. *sporogenes* strains PA 3679 (Bradbury et al., 2012) and ATCC 15579 in the UniProtKB database (The Uniprot consortium, 2011).

Major proteins identified from unwashed exosporium include likely soluble proteins which may be loosely associated, such as GroEL and several enzymes, and also a protein containing multiple 24 amino acid repeats, highly conserved in *C*. *botulinum* Group I. The protein IDs, and sequences and peptide identifications, are shown in Table 1 and SI-3.

The major proteins in 7 bands excised from the SDS-PAGE gel of washed exosporium were identified. Protein IDs are shown in Table 1 and sequences and peptide identifications are presented in SI-2. The very high molecular weight region (band 1) contained a 299 residue (25 cysteine) protein, which we named CsxA, (for *C*. *sporogenes* exosporium protein A) and a protein with a characteristic collagen-like repeat (CLR) domain as in *B*. *anthracis* BclA, which we named BclA by analogy; apart from the CLR domain, it is not a homologue of *B*. *anthracis* BclA. Band 2 (ca. 200 kDa) again contained BclA, along with a 151 residue (10 cysteine) protein, which we named CsxB. This latter protein was also found in bands 3, 4, 5, 6 and 7. These proteins in the high molecular weight bands are prime candidates to form structural components of the hairy nap and basal layer of the exosporium. Band 3 (ca. 60 kDa) also contained a carbon monoxide dehydrogenase, SpoIVA, and a LysM domain-containing protein. Band 4 (ca. 44 kDa) contained a number of potentially soluble proteins including for example a predicted aminotransferase, elongation factor Tu, glutamate dehydrogenase, and arginine deiminase, as well as CsxB. Band 5 (ca. 38 kDa), contained proteins annotated as aminotransferase, proline racemase, ornithine carbamoyltransferase, a pyruvate ferridoxin/flavodoxin oxidoreductase, and others, along with CsxB, a protein of the *B*. *subtilis* CotS family and a homologue of *B*. *subtilis* CotJC. Band 6 (ca. 30 kDa) identifications included CotJC, CotJB, a predicted 273 amino acid protein with 14 cys residues which we named CsxC and an NlpC/P60 cell wall peptidase family protein. In band 7 (ca. 19 kDa) CsxB, described above, was the sole identified component in this major band, which corresponds to the predicted size of the monomer.

To confirm the close similarity of *C*. *sporogenes* NCIMB 701792 to the sequenced strains, its *csxA* and *csxB* gene sequences were determined from PCR products of genomic DNA. The *csxA* gene and deduced protein sequence are both 100% identical to those of PA 3679, and those of *csxB* are 98% and 100% identical, respectively.

### 3.6 Protein profile of extracts from bulk *C*. *sporogenes* exosporium

*C*. *sporogenes* washed exosporium was incubated with detergent and the solubilized material was digested with trypsin. Sixty-eight proteins (Table 1 & SI-2), including CsxA, CsxB, CsxC and BclA, were identified. We also identified a second protein with a collagen-like domain; this has been named BclB as it has a C-terminal domain homologous (68% amino acid identity; Fig. S2) to that of BclB of *B*. *anthracis*.

### 3.7 Homologues in *C*. *botulinum*

**Fig. 5:**
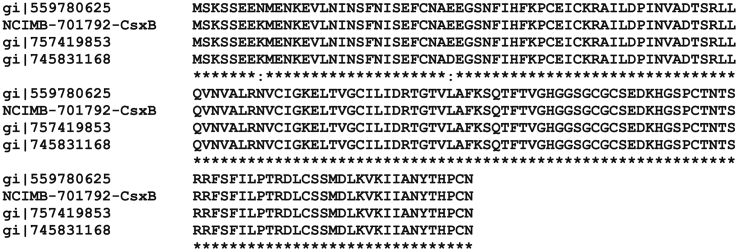
**Alignment of *C.* *sporogenes* NCIMB 701792 CsxB with *C*. *botulinum* homologues**. CsxB from *C*. *sporogenes* NCIMB 701792 is compared to: gi|559780625, *C*. *botulinum* B str. Osaka05; gi|757419853, *C*. *botulinum* B2 450 and gi|745831168, *C*. *botulinum* Prevot 594.

As so little is known of the exosporium in *Clostridia*, it is important to consider whether the major proteins identified in *C*. *sporogenes* are more widely distributed. The WGS database at NCBI was searched using BlastP. Homologues of the cysteine-rich proteins CsxA, CsxB and CsxC were found in *C*. *botulinum* Group I (Table 2). For example, CsxA is 100% conserved in *C*. *botulinum* strain Prevot 594 (Smith et al., 2015) and *C*. *sporogenes* PA 3679; homologues are present in other Group I strains, but are less conserved (78–87% identity; Table 2). *C*. *botulinum* strains in the *C*. *sporogenes* cluster share a near identical CsxB protein (99–100% identity), and a CsxB homologue (78% identity) is present in the other Group I cluster. The alignments of CsxA and CsxB with several *C*. *botulinum* homologues are shown in Fig. S3 and Fig. 5, respectively. CsxC is identical in *C*. *botulinum* strain Prevot 594, but is less closely conserved (61–77% identity) in other *C*. *botulinum* strains (Table 2). *C*. *botulinum* Eklund 17B was considered as a representative of the taxonomically distinct Group II (non-proteolytic) strains. It encodes a CsxA homologue (41% identity), but no homologues of CsxB and CsxC were detected. Homologues of CsxA and CsxB with lower amino acid identity (40%–30%) are present in a number of other *Clostridium* species, *including Clostridium tetani*, *Clostridium beijerinkii*, *Clostridium pasteurianum*, *Clostridium saccharoperbutylacetonicum*, *Clostridium senegalense*, *Clostridium kluyveri*, *Clostridium akagii and Clostridium tyrobutyricum*, but no CsxC homologues were detected in other *Clostridium* species. Of these species listed, a true exosporium has only been demonstrated in *C*. *pasteurianum* (Mackey and Morris, 1972) and *C*. *tyrobutyricum* (T K Janganan, unpublished data).

The BclA protein, identified in LC-MS/MS by its N-terminal peptide, is less widely distributed. The gene is present in *C*. *sporogenes* and in *C*. *botulinum* Prevot 594, but not in other Group I members; the next closest homologue (46% identity) is currently detected in *Clostridium scatologenes*. The BclB protein is detected in *C*. *sporogenes*, *C*. *botulinum* Prevot 594 (82% identity) and in *C*. *botulinum* strain 277-00 (Type B2), but is absent in other Type I strains. Other proteins with CLR domains are commonly present in the Group I genomes examined, including one with a C-terminal domain distantly related (28% identity) to the large N-terminal domain of BclA of *C*. *sporogenes*.

### 3.8 Comparative architecture of the exosporium

Our analysis of the spore surface of *C*. *sporogenes* confirms the presence of a hairy nap and a paracrystalline basal layer, as well as additional surface appendages, and identifies a number of proteins in purified exosporium. It revealed interesting surface features of *C*. *sporogenes* spores, especially a “beaded fibril” type (Fig. 3) that has not been reported before. Hairy nap and appendages are common surface features of the exosporium of *Clostridium* spores (Hodgkiss et al., 1967, Hoeniger and Headley, 1969), and hair like projections have been reported on the surface of *C*. *sporogenes* ATCC 3584 exosporium (Panessa-Warren et al., 1997). Multiple tubular appendages have been observed in some strains of type E *C*. *botulinum* (Hodgkiss et al., 1966). Exosporium with single appendages has been observed in *C*. *sporogenes* OS strain 24 AS (Hodgkiss et al., 1966). Brunt et al. (2015) have elegantly shown by scanning EM how the germinated cell emerges from an aperture at one pole of the exosporium of *C*. *sporogenes*. Some features of *C*. *sporogenes* exosporium resemble those of the *B*. *cereus* group, where the exosporium also appears as a deformable sac-like layer, extended at one spore pole and more tightly associated at the other end (Ball et al., 2008); again, there is a hairy nap, and a crystalline basal layer (Kailas et al., 2011).

### 3.9 Exosporium protein comparisons

Whilst the architecture of the outer surface of the *C*. *sporogenes* spore resembles that of the *B*. *cereus* group, the protein composition is notably different. Proteins from purified exosporium from *B*. *cereus* and *B*. *anthracis* include high molecular weight complexes on SDS-PAGE, composed of three primary constituents of the exosporium structure, BclA, ExsF/BxpB and ExsY. By analogy, the presence of BclA, CsxA and CsxB proteins in high molecular weight material in *C*. *sporogenes* exosporium extracts suggests that they may play a similar role in the *C*. *sporogenes* structure. In addition to BclA, a second CLR protein BclB has been detected in total exosporium. Similarly organised CLR proteins have been identified as components of the hairy nap in exosporium of *B*. *anthracis* (Sylvestre et al., 2003, Thompson et al., 2012). The N-termini of *B*. *anthracis* BclA and BclB both contain a motif responsible for their localisation to the exosporium (Thompson and Stewart, 2008, Thompson et al., 2012), but this motif is not present in the BclA or BclB proteins of *C*. *sporogenes*. BxpB/ExsFA has been shown to be a component of the exosporium in *B*. *anthracis* and acts as an anchor protein for BclA into the exosporium, but no identifiable homologue of the ExsF protein is encoded in the sequenced genome of any Clostridial species, including *C*. *sporogenes*. We do not know whether BclA and BclB are functionally equivalent to those in *B*. *anthracis*, but if indeed they are nap components, it is likely that they are linked to the basal layer by a different mechanism from that in *B*. *anthracis*.

We found a number of soluble and cytosolic proteins by LC-MS/MS, as also described in *B*. *cereus*, *B*. *anthracis* (Todd et al., 2003, Liu et al., 2004). Some of these proteins are detectable even after washing the exosporium extensively with salt and SDS (Table 1 & SI-1-3). This suggests that proteins might be associated with, or trapped in, the exosporium layer; some may be residual contaminating vegetative cell or spore coat proteins. The collection of proteins identified in exosporium may also include appendage proteins, as these are present in our exosporium preparation, although they would represent only a small proportion of the total protein.

## 4 Conclusion

This work provides the first insight into the likely composition and organization of the exosporium of Group I *C*. *botulinum* strains; our study has identified exosporium proteins with homologues across a wider range of *Clostridial* species, and provides the first level of information for more detailed study of the exosporium of these important bacteria. CsxA, CsxB and CsxC are potential candidates for structural proteins of the exosporium of *C*. *sporogenes* and *C*. *botulinum* and their contribution will be explored further. A fundamental understanding of exosporium structure and properties in *C*. *sporogenes* and *C*. *botulinum* will inform future studies of biological function and inactivation regimes.

## Figures and Tables

**Table 1:** Selected *C*. *sporogenes* proteins identified by LC-MS/MS analysis from bulk washed exosporium, and from SDS PAGE-separated proteins from washed and unwashed exosporium.

UniProtKB ID	MW (kDa)	Protein	Proteins identified from		
Washed exosporium gel bands-Fig. 4 & SI-1	Bulk washed exosporium-ID number in SI-2	Unwashed exosporium gel bands-Fig. S1 & Sl-3
G9F2G0	33.4	CsxA (25 cys)	band 1	ID 1	
J7T0S1	16.6	CsxB (10 cys)	band 2, 3, 4, 5, 6 & 7	ID 58	
G9F2F5	17.1	BclA fragment	band 1 & 2	ID 59	
G9F2J8	56.7	Stage ΙV A sporulation protein	band 3	ID 63	
G9EZ29	59.5	LysM domain containing protein	band 3		
G9F4A5	68.4	Carbon monoxide dehydrogenase	band 3	ID 60	
G9F1L8	44.9	Aminotransferase class I/II	band 4 & 5		
G9EWK8	43.4	Elongation factor Tu	band 4		
J7TAJ5	45.6	Glutamate dehydrogenase	band 4	ID 16	band 4
G9EYX8	46.5	Arginine deiminase	band 4	ID 15	
J7SU99	36.4	Proline racemase	band 5	ID 68	band 6
J7T8Q8	35.7	Ornithine carbamoyltransferase	band 5	ID 36	band 5
J7SXJ9	39.9	Pyruvate/ferridoxin/flavodoxin oxidoreductase	band 5		
J7SW79	42.5	CotS	band 5		
J7SGD1	21.4	Uncharacterised CotJC homologue	band 5 & 6	ID 67	
G9EW59	30	CsxC (14 cys)	band 6	ID 66	
G9EW78	21.3	CotJC	band 6	ID 64	
G9EW77	11.1	Spore coat peptide assembly protein CotJB	band 6	ID 65	
G9EWA8	35.4	NIpC/P60 family protein	band 6		
G9F4H9	23.8	BclB		ID 45	
J7T3Q4	6.6	Uncharacterised (4 cys)		ID 51	
G9EYZ3	44.4	Uncharacterised (7 cys)		ID 28	
G9EWT5	21.3	Uncharacterised (7 cys)		ID 31	
G9EWH5	8.4	Uncharacterised (12 cys)		ID 55	
G9F5J7	34.4	Stage III sporulation protein		ID 18	
J7TET6	26	Uncharacterised protein		ID 50	
G9EZQ2	60	Chaperonin GroEL		ID 62	band 3
G9F301	67.9	Peptidase M24		ID 37	band 2

**Table 2:** Homologues of *C*. *sporogenes* exosporium proteins in *C*. *botulinum* Group I and other *Clostridium* species.

Strain	CsxA	CsxB	CsxC
% Amino acid identity		
*C*. *botulinum*_Prevot 594	100	99	100
*C*. *botulinum* B2 450	87	100	77
*C*. *botulinum* Str. B Osaka05	86	99	77
*C*. *botulinum* A3 Loch Maree	77	78	65
*C*. *botulinum* F Str. Langeland	84	78	64
*C*. *botulinum* Hall	83	78	61
*C*. *tetani*	ND	43	ND
*C*. *beijerincki*	38	31	ND

^a^From WGS Blast (NCBI).

ND- No homologue detected.
