# Supplementary material for: Characterization of the spore surface and exosporium proteins of Clostridium sporogenes; implications for Clostridium botulinum group I strains
Source: Food Microbiol. 2016 Oct;59:205–12. doi: 10.1016/j.fm.2016.06.003 (PMC4942563; doi:10.1016/j.fm.2016.06.003)
Supplement: Supplementary file 1 [file mmc1.docx]

**Supplementary information**

**SI-1. Analysis of gel bands from *C. sporogenes***

Gel slices of 7 protein bands shown in Fig. 4 were subjected to LC-MS/MS. For each protein band of interest, spots of diameter 1.5 mm were excised from selected gel bands and transferred to a microtitre plate for trypsin digestion using a ProGest Investigator™ automated workstation (Genomic Solutions). Samples were digested according to the standard ProGest long trypsin protocol supplied with the instrument, using iodoacetamide for cysteine derivatisation. Peptides were analysed by nano-LC MS/MS using an Ultimate 3000 LC system (Dionex) linked to a Q-TOF mass spectrometer (QStar Pulsar i, AB SCIEX) via a nanospray source (Protana) with a PicoTip silica emitter (New Objective). Samples were loaded via a Zorbax 300SB-C18 5 x 0.3 mm column (Agilent) and peptides resolved using an Acclaim® PepMap 100 C18 column 75 µm x 15 cm (Thermo Scientific). A linear gradient of 5 to 32% ACN in 0.1% formic acid over 47 minutes at a flow rate of 300 nL/min was run and remaining peptides were then eluted with 72% ACN, 0.1% formic acid. MS and MS/MS data were acquired using Analyst QS software version 1.1, switching between 1 s survey and 3 x 3 s product ion scans during peptide elution. Ions with charge state 2+ to 4+ and TIC > 10 counts were selected for fragmentation. MS/MS data-files were processed with Protein Pilot software version 2.0.1(AB SCIEX), using the incorporated Paragon search algorithm for protein identification from a database containing 7995 *C. sporogenes* sequences downloaded from UniProtKB (search term ‘*Clostridium sporogenes’*), 33410 *A. thaliana* sequences (from TAIR10 release) and 162 common contaminant proteins supplied with the software. Search parameters were: sample type, ID; cys alkylation, iodoacetamide; digestion, trypsin; instrument QSTAR ESI; and a protein detection threshold score of 1.3 (95% confidence) as the minimum for protein identification to be included in the output. Data-files were also processed in a Paragon search with identical parameters against a decoy version of the database, where all of the individual protein sequences had been randomised.

The sequences of the highest-ranked identified proteins (or those with similar scores to the top-ranked) are shown, **with high confidence peptides in green, medium in yellow and low-confidence shown in red**. Several bands appeared to contain mixtures of proteins. Each peptide supported by spectral evidence with 99% confidence contributes a value of 2.0 (Peptide shown in green) to the protein score (under the header ‘total’). The percentage of sequence covered by peptide is shown under the header ‘% cov’. Those matched with less confidence contribute diminishing values to the total protein score.

The database UniProtKB (The UniProt Consortium., 2011) used contains entries from both *C. sporogenes* PA 3679 and ATCC 15579, which may increase the number of IDs. For conciseness, only one ID for each protein is shown below. Sequence identifications for the highest scoring proteins in each band are shown.

**Band 1 Proteins identified**

N Total %Cov UniProtKB Accession Number and description

1 9.27 36.8 G9F2G0_CLOSG Putative uncharacterized protein *C. sporogenes* PA 3679. **Named CsxA**

MAINSK**DFIPRPGFVNK**QGCLPDPVEICCIQVPKVFDQCLRKECLKPTDDCEQLCK**QIPNITDPSQVR**CVGCCK**NLKVIVNSVTK**CPVSNGKPGYKKVTINYTITFDVDVDVEINGVTQTQTLSYSVNR**TITASNLYCPDTIAK**TIIGK**ECTSAEEVDQQFIKIEVVGDCLSTDISK**IDCGGGCNCGCTCPDDPNNGDNKVFLCITLGLFIIIKCEIVVQLMVPAYGYCPVPEECKCSHDPCKEFMERELPTLYPPQEMDNLFDEYDERQDDRHIHNEKHIEEEEER**GNMITSSVITNN**

2 2.00 11.4 G9F2F5_CLOSG Collagen triple helix repeat protein (Fragment) *C. sporogenes* PA 3679. **Named BclA**

**MVNNSLQLQR**TTTGTIAANTNVIFDNTLLSTGSDISYNGGTGVVTITKTGIYYVDWWVTTQSSFSATYISFAIKTSDNKTIQGESPIKIGQVSGNALLNVTTVPYTFSLINSNLDVSLAVNPTVKANLSVTEETSTLGVTGPTGPQGITGATGPQGITGATGPQGV

**Band 2 Proteins identified**

N Total % Cov UniProtKB Accession Number and description

1 3.52 14.6 J7T0S1_CLOSG Uncharacterized protein *C. sporogenes* ATCC 15579. **Named CsxB**

MSKSSEEKMENKEVLNINSFNISEFCNAEEGSNFIHFKPCEICKR**AILDPINVADTSRLLQVNVALR**NVCIGKELTVGCILIDRTGTVLAFKSQTFTVGHGGSGCGCSEDKHGSPCTNTSRRFSFILPTRDLCSSMDLKVKIIANYTHPCN

N Total % Cov UniProtKB Accession No and description

2 2.17 11.4 G9F2F5_CLOSG Collagen triple helix repeat protein (Fragment) *C. sporogenes* PA 3679. **Named BclA**

**MVNNSLQLQR**TTTGTIAANTNVIFDNTLLSTGSDISYNGGTGVVTITKTGIYYVDWWVTTQSSFSATYISFAIKTSDNKTIQGESPIKIGQVSGNALLNVTTVPYTFSLINSNLDVSLAVNPTVKANLSVTEETSTLGVTGPTGPQGITGATGPQGITGATGPQGV

**Band 3 Proteins identified**

N Total % Cov UniProtKB Accession No and description

1 16.44 33.6 G9F4A5_CLOSG Carbon monoxide dehydrogenase *C. sporogenes* PA 3679

2 6.68 16.6 G9F3V1_CLOSG V-type ATP synthase alpha chain *C. sporogenes* PA 3679

3 5.09 18.7 G9EZ29_CLOSG LysM domain-containing protein *C. sporogenes* PA 3679

4 4.36 16.2 G9F4E5_CLOSG Phosphoglucomutase/phosphomannomutase family protein *C. sporogenes* PA 3679

5 2.88 15.5 G9EZQ2_CLOSG 60 kDa chaperonin OS= *C. sporogenes* PA 3679

6 2.00 7.4 G9F3C8_CLOSG Glutamine--fructose-6-phosphate aminotransferase [isomerizing] *C. sporogenes* PA 3679

7 2.00 11.0 G9F2J8_CLOSG Stage IV sporulation protein A *C. sporogenes* PA 3679

9 1.71 14.6 J7T0S1_CLOSG Uncharacterized protein *C. sporogenes* ATCC 15579. **Named CsxB**

10 1.40 6.9 G9F1I6_CLOSG Oligoendopeptidase F *C. sporogenes* PA 3679

**Top scoring protein:**

N Total % Cov UniProtKB Accession No and description

1 16.44 33.6 G9F4A5_CLOSG Carbon monoxide dehydrogenase OS=Clostridium sporogenes PA 3679

MSMCTNCKTCKSADKVLQGFISNMDMETSHHRVEDQKIKCGFGQLGVCCRLCANGPCRITPKSPR**GVCGASADTIVAR**NFLR**AVAAGAGCYLHIVENTAR**NLKATGENKGKIKGEKALNR**LADIFEIK**EEDIYKKAVRVADMVLKDLYKPRYEKMEIVEKMAYKPRYENWEKLNILPGGAKSEVFDAVVKTSTNLNSDPVEMLLHCLNLGISTGLYGLTLTNLLNDVMLGEPVIRPAKVGFRVIDESYINIMITGHQHSTIAHLQDRLIDKDIVEMAKKVGAKGFR**LVGCTCVGQDLQLR**GEHYQEVFSGHGGNNFTSEAIIATGAIDAIVSEFNCTLPGIEPITENLKVKMICLDDVAKKSNAEYLEYSYEHREKISEHIIKEALDAYKERRKNIKVNIPR**DHGYNDVITGVSEVSLK**EFLGGTWKPLLDLISEGKIKGVAGVVGCSNLTAMGHDVFTVELTKELIKRDIIVLSAGCSSGGLENVGLMSTSAASLAGDNLRAVCESLKIPPVLNFGPCLAIGR**LEIVATELAK**DLGIDLPQLPLVLSAPQWLEEQALADGAFGLALGLPLHLAISPFIGGSK**VVSKVLTEDLKTLTGGQLIIEDDVK**K**AADKLEAIVLDR**REKLGLK

**Band 4 Proteins identified**

N Total % Cov UniProtKB Accession No and description#

1 11.26 36.1 G9F1L8_CLOSG Aminotransferase, classes I and II *C. sporogenes* PA 3679

2 7.31 17.4 G9EWK8_CLOSG Elongation factor Tu *C. sporogenes* PA 3679

3 6.60 26.6 J7TAJ5_CLOSG Glutamate dehydrogenase *C. sporogenes* ATCC 15579

4 6.33 26.0 J7T0J8_CLOSG Aminotransferase, class I/II *C. sporogenes* ATCC 15579

5 5.70 15.9 G9EYX8_CLOSG Arginine deiminase *C. sporogenes* PA 3679

6 5.52 23.0 J7T4T3_CLOSG 3-methyl-2-oxobutanoate dehydrogenase (2-methylpropanoyl-transferring) *C. sporogenes* ATCC 15579

7 4.48 16.2 J7SZM3_CLOSG Serine hydroxymethyltransferase *C. sporogenes* ATCC 15579

8 2.06 11.3 J7T7W9_CLOSG Phosphomethylpyrimidine synthase *C. sporogenes* ATCC 15579

9 2.00 17.5 J7T1S2_CLOSG ATP synthase subunit alpha *C. sporogenes* ATCC 15579

10 2.00 9.0 J7T7J6_CLOSG S-adenosylmethionine synthase *C. sporogenes* ATCC 15579

11 2.00 3.5 G9EZR4_CLOSG Electron transfer flavoprotein, alpha subunit/FixB family protein *C. sporogenes* PA 3679

12 1.70 9.1 J7T006_CLOSG R-phenyllactate dehydratase, small subunit *C. sporogenes* ATCC 15579

13 1.52 8.6 J7T0S1_CLOSG Uncharacterized protein *C. sporogenes* ATCC 15579. **Named CsxB**

**Top scoring protein:**

N Total % Cov UniProtKB Accession No and description

1 11.26 36.1 G9F1L8_CLOSG Aminotransferase, classes I and II *C. sporogenes* PA 3679

MNIRFSER**AAGLKASEIR**ELLKLTEMPEIISFAGGLPAPELFPVEEMK**GIMQEVLDTQGRAALQYSSTEGYKPLR**EIIANERMKPAGVNVSYENIAITNGSQQGIEFSAKIFLNEGDIVVCESPSYLGAINAFKSYRPKFVEIPMDDNGMIIEELEKALAENKGKVK**MIYTIPDFQNPTGR**TMPDDRRKRIAELAAEYEIPVIEDNPYGDLIYEGERHPSIKSFDKEGWVIYLGTFSKNFCPGLRLAWVCAEPEILDKYIIVK**QGVDLQAGTLDQR**ATALFMQK**YDLNEHIEK**IKKVYEKRRDLMLDSMKKYFPAGVKYTHPVGGLFTWVELREDLDAKELMKDALAENVAYVPGGSFFPNGGHENYFRLNYSCMSDEKIVEGVKRLGKVLDKYYK

**Band 5 Proteins identified**

N Total % Cov UniProtKB Accession No and description #

1 6.11 19.2 J7TAK9_CLOSG Aminotransferase, class I/II *C. sporogenes* ATCC 15579

2 5.91 23.0 J7SU99_CLOSG Proline racemase *C. sporogenes* ATCC 15579

3 5.45 36.4 J7T8Q8_CLOSG Ornithine carbamoyltransferase *C. sporogenes* ATCC 15579

4 4.51 20.5 J7SXJ9_CLOSG Pyruvate flavodoxin/ferredoxin oxidoreductase, thiamine diP-binding domain protein *C. sporogenes* ATCC 15579

5 4.32 16.9 G9F208_CLOSG Electron transfer flavoprotein, alpha subunit/FixB family protein *C. sporogenes* PA 3679

6 4.03 17.9 G9EWK8_CLOSG Elongation factor Tu *C. sporogenes* PA 3679

7 3.73 19.2 J7SXE6_CLOSG Glyceraldehyde-3-phosphate dehydrogenase, type I *C. sporogenes* ATCC 15579

8 3.10 26.5 J7T0S1_CLOSG Uncharacterized protein *C. sporogenes* ATCC 15579. **Named CsxB**

9 2.25 18.4 J7SHB8_CLOSG 4-phosphoerythronate dehydrogenase *C. sporogenes* ATCC 15579

10 2.17 6.2 J7SH15_CLOSG Branched-chain-amino-acid aminotransferase *C. sporogenes* ATCC 15579

11 2.16 23.8 J7T3B5_CLOSG Cell shape determining protein, MreB/Mrl family *C. sporogenes* ATCC 15579

12 2.00 10.9 G9F3Y4_CLOSG Serine hydroxymethyltransferase *C. sporogenes* PA 3679

13 2.00 7.5 J7SW79_CLOSG Spore coat protein, CotS family *C. sporogenes* ATCC 15579

14 2.00 7.3 J7SFL9_CLOSG Ornithine cyclodeaminase *C. sporogenes* ATCC 15579

15 2.00 5.8 J7SGD1_CLOSG Uncharacterized protein (CotJC) *C. sporogenes* ATCC 15579

16 1.70 2.7 J7T4T3_CLOSG 3-methyl-2-oxobutanoate dehydrogenase (2-methylpropanoyl-transferring) *C. sporogenes* ATCC 15579

17 1.52 12.5 G9F0U9_CLOSG NlpC/P60 family protein *C. sporogenes* PA 3679

**Top scoring proteins:**

N Total % Cov UniProtKB Accession No and description

1 6.11 19.2 J7TAK9_CLOSG Aminotransferase, class I/II *C. sporogenes* ATCC 15579 and

6.11 19.2 G9F1L8_CLOSG Aminotransferase, classes I and II *C. sporogenes* PA 3679

MLFNDKLRQNIMRKYDKILTSMGFFKNFFDFLYIEKKDIISVCRIDISIKVLINLLYFWRDLLMNIRFSERASGLKASEIRELLKLTEMPEIISFAGGLPAPELFPVEEMKGIMQEVLDTQGR**AALQYSSTEGYKPLR**EIIANERMKPAGVNVSYENIAITNGSQQGIEFSAKIFLNEGDIVVCESPSYLGAINAFKSYRPKFVEIPMDDNGMIIEELEKALAENKGKVK**MIYTIPDFQNPTGR**TMPDDRRKRIAELAAEYEIPVIEDNPYGDLIYEGERHPSIKSFDKEGWVIYLGTFSKNFCPGLRLAWVCAEPEILDKYIIVK**QGVDLQAGTLDQR**ATALFMQKYDLNEHIEKIKKVYEKRRDLMLDSMKKYFPAGVKYTHPVGGLFTWVELREDLDAKELMKDALAENVAYVPGGSFFPNGGHENYFRLNYSCMSDEKIVEGVKRLGKVLDKYYK

N Total % Cov UniprotKB Accession No and description

2 5.91 23 J7SU99_CLOSG Proline racemase, *C. sporogenes* ATCC 15579

MRAIKTIQTIESHTMGEPTRIVIGGLPKVPGKTMAEK**MEYLEENNDSLR**TMLMSEPRGHNDMFGAIYTEPADETADLGIIFMDGGGYLNMCGHGSIGAATCAVEMGIVKVEEPYTNIKLEAPAGMINARVKVEDGKAKETSIVNVPAFLYKK**DVEIDVPDYGK**LTLDISFGGSFFAMVDAEK**VGIDISPANSQK**LNDLGMK**IVHAVNEQVEIK**HPVLEHIKTVDLCEFYGPAKSEDADVQNVVVFGQGQVDRSPCGTGTSAKMALLYAQGKMKVGEEIVNESIICTKFKGKILEETKVGEYDGIIPEITGSAYVTGFSQFLVDEEDPVKYGFVLK

**Band 6 Proteins identified**

N Total % Cov UniProtKB Accession No and description

1 10.36 44.7 G9EW78_CLOSG CotJC protein *C. sporogenes* PA 3679

2 6.46 25.8 J7T0S1_CLOSG Uncharacterized protein *C. sporogenes* ATCC 15579. **Named CxsB**

3 5.45 36.7 G9EW77_CLOSG Spore coat peptide assembly protein CotJB *C. sporogenes* PA 3679

4 4.11 16.5 G9EWA8_CLOSG NlpC/P60 family protein *C. sporogenes* PA 3679

5 2.74 7.7 G9EW59_CLOSG Putative uncharacterized protein *C. sporogenes* PA 3679. **Named CsxC**

6 6.31 34.2 J7SGD1_CLOSG Uncharacterized protein, cotJC homologue *C. sporogenes* ATCC 15579

7 2.00 2.8 J7TAK9_CLOSG Aminotransferase, class I/II *C. sporogenes* ATCC 15579

8 1.32 12.0 J7TFI4_CLOSG 4-hydroxy-tetrahydrodipicolinate synthase *C. sporogenes* ATCC 15579

**Top scoring proteins:**

N Total %cov UniProtKB Accession

1 10.36 44.7 G9EW78_CLOSG CotJC protein *C. sporogenes* PA 3679 and

6 6.31 34.2 J7SGD1_CLOSG Uncharacterized protein *C. sporogenes* ATCC 15579

MWIYEKKLEYPVNLKSKDLGMAK**FLMAQYGGPDGELSAALR**YLSQRYTMPTSKSKGLLTDIGTEELAHVEIIATMVYQIMENATPKELREAGLGSYYTEHGNAIYPADANGIPWTAAYIQSMAD**PITDLHEDMAAEQKAR**TTYEHLMNLTDDHDIKDVLAFLRQREVVHFQR**FGEALMSVEDKVNSR**TYY

N Total % Cov UniProtKB Accession No and description

2 6.46 25.8 J7T0S1_CLOSG Uncharacterized protein *C. sporogenes* ATCC 15579. **Named CsxB**

2 6.46 25.8 G9EY44_CLOSG Putative uncharacterized protein *C. sporogenes* PA 3679. **Named CsxB**

MSKSSEEKMENKEVLNINSFNISEFCNAEEGSNFIHFKPCEICKR**AILDPINVADTSR**LLQVNVALRNVCIGKELTVGCILIDR**TGTVLAFK**SQTFTVGHGGSGCGCSEDKHGSPCTNTSRR**FSFILPTR**DLCSSMDLKVK**IIANYTHPCN**

N Total % Cov UniProtKB Accession and description

3 5.45 36.7 G9EW77_CLOSG Spore coat peptide assembly protein CotJB *C. sporogenes* PA 3679

MLDKCSRKELLFMIQQYEFTAVELNLYLDNYPDNKK**ALEYYNEISCKLEELIETYEKRFGPLFNFGFSK**SKFPWQWTTQPWPWENEYSRM

N Total % Cov UniProtKB Accession #

5 2.74 7.7 G9EW59_CLOSG Putative uncharacterized protein *C. sporogenes* PA 3679. **Named CsxC**

MSMDEMRGNYDSNSYKSGNYDCHKDCGKVIESKTLPLCDGTDITPETVAPPVVAKIPVVIAEQEIQVDVEARMKLKEKYYEIKRIRKDVFLTQCELLPR**AGVIEDGVPVTGK**LFISGYVKKNIEYATADCVKHDAVSGDIKHTTEKIPFNCVTEVTYITPPIVSNRGIQRRTDLYCDEGLCDCSCREEKLGKLNCQEYLEDVVTYVEKPYCELMGAR**IFETDIQR**KPCYEDGVKVYDELLEKMVVYVRVKVLQLQQVAIDNGAGGLGCRSKEH

**Band 7 Proteins identified**

N Total % Cov UniProtKB Accession No and description

1 9.13 25.8 J7T0S1_CLOSG Uncharacterized protein OS= *C. sporogenes* ATCC 15579. **Named CsxB** and

1 9.13 25.8 G9EY44_CLOSG Putative uncharacterized protein *C. sporogenes* PA 3679. **Named CsxB**

MSKSSEEKMENKEVLNINSFNISEFCNAEEGSNFIHFKPCEICKR**AILDPINVADTSR**LLQVNVALRNVCIGKELTVGCILIDR**TGTVLAFK**SQTFTVGHGGSGCGCSEDKHGSPCTNTSRR**FSFILPTRDLCSSMDLKVKIIANYTHPCN**

**SI-2. Analysis of *C. sporogenes* bulk exosporium proteins**

Washed exosporium (40 μg protein) was resuspended at 1 mg/ml in 0.5% deoxycholic acid, 12 mM N-lauroylsarcosine, 50 mM ammonium bicarbonate and incubated for 2 x 10 min at 50°C, interspersed with disruption for 5 min in a sonicating water-bath. The insoluble material was removed by centrifugation at 40,000 x*g* for 20 min and discarded. Supernatant only was taken for subsequent analysis. Cysteine residues were modified by treatment with 12.5 mM DTT at 56°C for 20 min, followed by addition of 25 mM iodoacetamide and incubation for 20 min at room temperature. The sample was diluted to 0.1 mg/ml in 50 mM ammonium bicarbonate and digested overnight at 37°C with 2 µg sequencing grade modified trypsin (Promega). The digest was freeze-dried and the residue re-suspended in 2% acetonitrile, 0.1% formic acid for nano-LC-MS/MS analysis. Data acquisition and analysis was performed as for the gel-bands. Peptide sequences matched to spectra with high confidence are shown in green, medium confidence in yellow and low-confidence (non-scoring) peptides in red. A peptide sequence identified with 99% confidence contributes a score of 2 to the total protein score. Possible structural proteins are shown in bold. Selected sequences are included with peptide identification at the end of the table.

**N Total % Cov UniProtKB Accession No and description**

**1 12.61 32.8 G9F2G0_CLOSG CsxA (25 cysteines); Putative uncharacterized protein *C. sporogenes*** **PA 3679**

2 11.54 16.5 G9F3G8_CLOSG Pyruvate-flavodoxin oxidoreductase *C. sporogenes* PA 3679

3 8.23 27.7 G9EWK8_CLOSG Elongation factor Tu *C. sporogenes* PA 3679

4 8.00 13.9 J7TB00_CLOSG Putative formate C-acetyltransferase *C. sporogenes* ATCC 15579

5 8.00 27.8 G9F1L8_CLOSG Aminotransferase, classes I and II *C. sporogenes* PA 3679

6 6.23 26.8 G9F134_CLOSG Putative electron transport protein *C. sporogenes* PA 3679

7 6.00 14.4 J7T1J1_CLOSG Peptidase, M24 family *C. sporogenes* ATCC 15579

8 4.91 17.4 G9F1M0_CLOSG Aspartate aminotransferase *C. sporogenes* PA 3679

9 4.24 16.3 J7SXH3_CLOSG Elongation factor G *C. sporogenes* ATCC 15579

10 4.15 22.0 J7SH15_CLOSG Branched-chain-amino-acid aminotransferase OS= *C. sporogenes* ATCC 15579

11 4.00 15.4 G9F2G7_CLOSG Glutamate decarboxylase *C. sporogenes* PA 3679

12 4.00 14.5 G9F4E5_CLOSG Phosphoglucomutase/phosphomannomutase family protein *C. sporogenes* PA 3679

13 4.00 20.5 G9EVH5_CLOSG Electron transport complex, RnfABCDGE type, C subunit *C. sporogenes* PA 3679

14 4.00 14.8 G9F359_CLOSG 50S ribosomal protein L2 *C. sporogenes* PA 3679

15 4.00 12.9 G9EYX8_CLOSG Arginine deiminase *C. sporogenes* PA 3679

16 4.00 13.3 J7TAJ5_CLOSG Glutamate dehydrogenase *C. sporogenes* ATCC 15579

17 4.00 19.9 J7SXI6_CLOSG 30S ribosomal protein S4 *C. sporogenes* ATCC 15579

18 4.00 16.9 G9F5J7_CLOSG Stage III sporulation protein AA *C. sporogenes* PA 3679

19 4.00 6.6 J7T2X1_CLOSG Aminotransferase, class I/II *C. sporogenes* ATCC 15579

20 3.70 13.7 J7SU43_CLOSG V-type ATP synthase beta chain *C. sporogenes* ATCC 15579

21 3.70 5.1 G9F0U9_CLOSG NlpC/P60 family protein *C. sporogenes* PA 3679 GN=IYC_10489 PE=4 SV=1

22 3.40 19.0 J7SHB8_CLOSG 4-phosphoerythronate dehydrogenase *C. sporogenes* ATCC 15579

23 3.40 6.7 J7T006_CLOSG R-phenyllactate dehydratase, small subunit *C. sporogenes* ATCC 15579

24 3.22 12.9 J7SZL9_CLOSG M18 family aminopeptidase *C. sporogenes* ATCC 15579

25 2.92 15.5 J7SZM3_CLOSG Serine hydroxymethyltransferase *C. sporogenes* ATCC 15579

26 2.71 13.3 J7SZ59_CLOSG Pyruvate, phosphate dikinase *C. sporogenes* ATCC 15579

27 2.49 17.6 J7SUB3_CLOSG 30S ribosomal protein S2 *C. sporogenes* ATCC 15579

**28 2.44 12.2 G9EYZ3_CLOSG Putative uncharacterized protein *C. sporogenes*** **PA 3679 (7 cysteine residues)**

29 2.03 29.1 J7SGC6_CLOSG Selenoprotein B, glycine/betaine/sarcosine/D-proline reductase family *C. sporogenes* ATCC 15579

30 2.02 15.3 J7SW42_CLOSG Fructose-1,6-bisphosphate aldolase, class II *C. sporogenes* ATCC 15579

**31 2.01 17.0 G9EWT5_CLOSG Putative uncharacterized protein *C. sporogenes*** **PA 3679 (7 cysteine residues)**

32 2.00 9.4 G9EZN9_CLOSG Peptidase family protein *C. sporogenes* PA 3679

33 2.00 9.1 G9EVM8_CLOSG DNA-directed RNA polymerase subunit beta' *C. sporogenes* PA 3679

34 2.00 20.7 J7T176_CLOSG Glycine reductase complex component C, beta subunit *C. sporogenes* ATCC 15579

35 2.00 8.9 J7TE55_CLOSG Dihydroxyacetone kinase, DhaK subunit *C. sporogenes* ATCC 15579

36 2.00 15.1 G9F304_CLOSG Aspartate/ornithine carbamoyltransferase family protein *C. sporogenes* PA 3679

37 2.00 17.3 G9F301_CLOSG Peptidase *C. sporogenes* PA 3679

38 2.00 37.7 J7T875_CLOSG Flavodoxin *C. sporogenes* ATCC 15579

39 2.00 7.9 J7T7J6_CLOSG S-adenosylmethionine synthase *C. sporogenes* ATCC 15579

40 2.00 9.2 |J7T4T3_CLOSG 3-methyl-2-oxobutanoate dehydrogenase (2-methylpropanoyl-transferring) *C. sporogenes* ATCC 15579

41 2.00 12.1 J7T362_CLOSG DNA-directed RNA polymerase subunit alpha *C. sporogenes* ATCC 15579

42 2.00 7.2 J7T134_CLOSG Carbon-monoxide dehydrogenase, catalytic subunit *C. sporogenes* ATCC 15579

43 2.00 15.1 J7SXE6_CLOSG Glyceraldehyde-3-phosphate dehydrogenase, type I *C. sporogenes* ATCC 15579

44 2.00 14.5 J7SFN9_CLOSG Respiratory-chain NADH dehydrogenase 51 kDa subunit *C. sporogenes* ATCC 15579

**45 2.00 20.9 G9F4H9_CLOSG BclB; Exosporium protein *C. sporogenes*** **PA 3679**

46 2.00 16.6 G9F3E1_CLOSG 2-ketoisovalerate ferredoxin reductase *C. sporogenes* PA 3679

47 2.00 15.2 G9F245_CLOSG NAD-binding Rossmann fold family oxidoreductase *C. sporogenes* PA 3679

48 2.00 10.1 G9EY30_CLOSG tRNA uridine 5-carboxymethylaminomethyl modification enzyme GidA C. sporogenes PA 3679

49 2.00 10.3 G9EWT2_CLOSG Pantothenate synthetase *C. sporogenes* PA 3679

50 2.00 5.3 J7TET6_CLOSG Uncharacterized protein *C. sporogenes* ATCC 15579

**51 2.00 23.6 J7T3Q4_CLOSG Uncharacterized protein *C. sporogenes* ATCC 15579 (4 cys residues)**

52 2.00 3.8 G9F2Y5_CLOSG Aminoacyl-histidine dipeptidase *C. sporogenes* PA 3679

53 2.00 3.0 G9EZR4_CLOSG Electron transfer flavoprotein, alpha subunit/FixB family protein *C. sporogenes* PA 3679

54 1.70 3.2 J7SFT4_CLOSG Acyl-CoA dehydrogenase, C-terminal domain protein *C. sporogenes* ATCC 15579

**55 1.70 13.2 G9EWH5_CLOSG Putative uncharacterized protein *C. sporogenes* PA 3679 (12 cys residues)**

56 1.52 9.7 J7SXH7_CLOSG 50S ribosomal protein L16 *C. sporogenes* ATCC 15579

57 1.51 8.0 G9EXB8_CLOSG ATP-dependent protease *C. sporogenes* PA 3679

**58 3.52 14.6 J7T0S1_CLOSG CsxB; uncharacterised protein *C. sporogenes* ATCC15579.**

**59 2.17 11.4 G9F2F5_CLOSG BclA; collagen triple helix repeat protein (Fragment) *C. sporogenes* PA 3679.**

60 16.44 33.6 G9F4A5_CLOSG Carbon monoxide dehydrogenase *C. sporogenes* PA 3679

61 6.68 16.6 G9F3V1_CLOSG V-type ATP synthase alpha chain *C. sporogenes* PA3679

62 2.88 15.5 G9EZQ2_CLOSG 60 kDa chaperonin *C. sporogenes* PA 3679

63 2 11 G9F2J8_CLOSG Stage IV sporulation protein A *C. sporogenes* PA 3679

64 10.36 44.7 G9EW78_CLOSG CotJC protein *C. sporogenes* PA 3679

65 5.45 36.7 G9EW77_CLOSG Spore coat peptide assembly protein cotJB *C. sporogenes*

66 2.74 7.7 G9EW59_CLOSG Putative uncharacterized protein *C. sporogenes*

67 6.31 34.2 J7SGD1_CLOSG Uncharacterized protein CotJC homologue *C. sporogenes*

68 5.91 23 J7SU99_CLOSG Proline racemase *C. sporogenes* ATCC 15579

**Amino acid sequences of selected protein IDs and peptides identified**

**Protein ID 1:** Putative uncharacterized protein *C. sporogenes* PA 3679 **(CsxA)**

M**AINSKDFIPRPGFVNKQGCLPDPVEICCIQVPK**VFDQCLR**KECLKPTDDCEQLCK**QIPNITDPSQVRCVGCCKNLKVIVNSVTKCPVSNGKPGYKKVTINYTITFDVDVDVEINGVTQTQTLSYSVNRTITASNLYCPDTIAKTIIGKECTSAEEVDQQFIKIEVVGDCLSTDISKIDCGGGCNCGCTCPDDPNNGDNKVFLCITLGLFIIIKCEIVVQLMVPAYGYCPVPEECK**CSHDPCKEFMER**ELPTLYPPQEMDNLFDEYDERQDDRHIHNEKHIEEEEERGNMITSSVITNN

**Protein ID 2:** Pyruvate-flavodoxin oxidoreductase *C. sporogenes* PA 3679

MRRMKTMDGNTAAAYISYAFTDVAAIYPITPSSPMAEHVDEWVAQGKKNIFGQPVKVMEMQSEAGAAGAVHGSLQAGALTTTYTASQGLLLMIPNMYK**IAGELLPGVFHVSAR**ALAANSLNIFGDHQDVMAARQTGLALLAESSVQQVMDLSAVAHLSAIEGRVPFINFFDGFRTSHEIQKVEVLEYDELENLVDMDGVKAFRRRALNPDHPVIRGTAQNPDIYFQEREVSNNYYERLPEIVEKYMGEISKLTGREYHLFNYYGAEDAERLIIAMGSVCDTVEEVVDYLMAKGEKVGLLTVHLYRPFSLEHFFKYIPKTVKNIAVLDR**TKEPGALAEPLYLDVK**NAFYGKEWQPTIVGGRYGLGSK**ETYPSHILSVYENLK**KDEPKDGFTIGIVDDVTNTSLEESEAINTTPAGTTACKFWGLGSDGTVGANKSAIK**IIGDHTDMYAQGYFAYDSK**KSGGITISHLRFGKSPIQSPY**LINQADFVACHNQSYVYK**YNVLEGLKKGGRFLLNTIWTPEEVETHLPASMKKYIAENDIEFYTLNAVKIAQEIGLGGRINMICQAAFFKIANIIPVEDAVKYLKDAVVTNYGKKGQKIIDMNNAAIDKGVNAIVKIEVPASWKDAKCEGSCEAKENPEFIKNIVEPMNRQEGDKLPVSAFKGMEDGTFPSGTAAYEKRGIAINVPEWQLDKCIQCNQCSYVCPHAVIRPVLLSDEEVKNAPEGFKSKPAVGAKGLNFTMAISPYDCTGCGNCADVCPAKEKALIMKPFDTQIEQDKNWEYAMKVSPKANPMKKNSVKGSQFEQPLLEFSGACAGCGETPYAKLVTQLFGDRMMIANATGCSSIWGASAPSTPYTTNHKGYGPAWANSLFEDNAEFGMGMYLGVKQIRDKVTEDVKAVLGFKSAEELQSCAIGTEDCSEKDMTGTVISGELRAALEDWLNNKDLGEGTRERADKVIELVGKEKGSDKFLNEIYENKDFLVKRSHWIFGGDGWAYDIGYGGVDHVLASGEDVNILVFDTEVYSNTGGQSSKATPTAAIAKFAASGKKTKKKDLGAMAMTYGYVYVAQIAMGADKNQTLKAIAEAEAYPGPSLIIAYAPCINHGLKAGMGCSQLEEKK**AVDCGYWGLYR**FNPELKEAGKNPFSLDSKEPTANFKDFLMGEVRYASLAKQFPEDAEALFAKTEQDAKERLENYKKLAEQ

**Protein ID 3:** Elongation factor Tu *C. sporogenes* PA 3679

MAKAKFERSKPHVNIGTIGHVDHGKTTLTAAITTVLAQKGGASATKYDEIDKAPEEKERGITINTSHVEYETANR**HYAHVDCPGHADYVKNMITGAAQMDGAILVVSAADGPMPQTR**EHILLASRVGVQYIVVFLNKADQVDDPELIELVEMEVRELLNEYGFPGDDTPIVVGSALEVLENQDNAEKTKCIDELMEAIDSYIPTPERATDQPFLMPVEDVFTITGRGTVATGRVER**GVLHTGDEVELIGMK**EEISKTVCTGIEMFRK**ILDEAMAGDNIGALLR**GVQRDDIQRGQVLAKPGSVTPHKKFVGQVYVLKKEEGGRHTPFFNGYRPQFYFRTTDVTGSINLPEGVEMVMPGDHIDMAVELITPVAMHENLRFAIREGGRTVGSGVVTTISE

**Protein ID 5:** Aminotransferase, classes I and II *C. sporogenes* PA 3679

MNIRFSERAAGLKASEIRELLKLTEMPEIISFAGGLPAPELFPVEEMKGIMQEVLDTQGR**AALQYSSTEGYKPLR**EIIANER**MKPAGVNVSYENIAITNGSQQGIEFSAK**IFLNEGDIVVCESPSYLGAINAFKSYRPKFVEIPMDDNGMIIEELEKALAENKGKVK**MIYTIPDFQNPTGR**TMPDDRRKRIAELAAEYEIPVIEDNPYGDLIYEGERHPSIKSFDKEGWVIYLGTFSKNFCPGLR**LAWVCAEPEILDK**YIIVKQGVDLQAGTLDQRATALFMQKYDLNEHIEKIKKVYEKRRDLMLDSMKKYFPAGVKYTHPVGGLFTWVELREDLDAKELMKDALAENVAYVPGGSFFPNGGHENYFRLNYSCMSDEKIVEGVKRLGKVLDKYYK

**Protein ID 7:** Peptidase, M24 family *C. sporogenes* ATCC 15579

MNQERLNKVLEGMKEREIPQMLISDAPAIFYLTGKWIHTGERLIALYLNENGNHKLFINELFPVTEDLGVEK**VWFNDNQDGVEIISK**YVEKDKVMGVDKNWPARFLLRLMELEGGSKFVNGSIIIDRARMFKDEKEKELMRASSKANDAAMEKLYSLFKENQDLSEKEVGERLAKIYSDLGAERFSFDPIVGYGANAADPHHENDGSK**LKEGDCIVLDIGCVK**DSYCSDMTRVFFYKSVPEHSK**EVYDTVVAANMAGIAAVKPGVR**FCDIDKASRDVIEKAGYGKYFTHRTGHSIGIEDHDLGDVSAVNTEEIKPGMIFSIEPGIYLPGEVGVRIEDLVLVTEDGCEVLNKYSKELTIIE

**Protein ID 18**: Stage III sporulation protein *C. sporogenes* PA 3679

MYTKEILNILPSHISRLICDLDEVDKLQEIRFKIGKPICFQIGNKEKLASYEVKREDIKSIVQRMSNYSIYSFEEEIKQGYLTIKGGHRVGICGRCVIDGGKVKTIRDISSLNIRICREIYNASKLVMPYIVENGQVLNTIIISPPKCGKTTIIRDISKKISDGVDSLNLKGQKVSVIDERSEIAGSYNGVPQLDVGLRTDVLDNCPKSEGIVMAIRSMAPEVIICDEIGTYKDVESILIALNSGVSLITTIHGFGVEDIYNRPVFKEIVENKVFKRAIVLSSKKSVGTLEYVYDFNKKTKLYCRII

**Protein ID 28:** Putative uncharacterized protein *C. sporogenes* PA 3679

G9EYZ3_CLOSG *C. sporogenes* PA 3679

MAQKKNYSR**YFIILEEDEK**GYSLGVDKSASGYVKLENKNGKCKISYYVQNIKKQSSPYYMVLICNKKGSKDIIKIGEMNIDEYGRADICYEYPVDNIGNCGINADKISGAAIVKFLDSNIISVMSGFSTTDIPAWKSFSIIESKERKKEDIKEEKTNKTIFDKYEETIEEIKIKDSNNTNKDGDNKVKSVQEHKEKGKDIKESNEKDLGSEDRKKGKDIKESDEKDLGKTKKPIEKNEYKKRNKENTPIGLEGKYFRSLVEDLDSVDNITDEIKNCIWYKIDAKDEDDMRNTCNYDKFMVLYNPMLGYYSYIKKHGHYILGYKCDSSGNMKYLVYGIPGDKTREDQPLKGK**SGFVTWIENK**ENNLGYWLMFYDYKTNNILIPVK

J7SGM7_CLOSG *C. sporogenes* ATCC 15579

MAQKKNYSR**YFIILEEDEK**GYSLGVDKSASGYVKLENKNGKCKISYYVQNIKKQSSPYHMVLICNKKGSKDIIKIGEMNIDEYGRADICYEYPVDNIGNCGINADKISGAAIVKFLDSNIISVMSGFSTTDIPAWKSFSIIESKERKKEDIKEEKVNKTIFDKYEETIEEIKTKDNNTNKDGDNKVKSVQEHKEKNLHSENREKGKDIKESNGKDLDSEDREKGRDIKESNGKDLDSEDREKDKNIKESDEKDLDSEDREKGKDIEKSNEKDLEKTKKPIGKNEYKKRNKQNTPIGLEGKYFRSLVEDLDSVDNITDEIKNCIWYKIDAKDEDDMRNTCNYDKFMVLYNPMLGYYSYIKKHGHYILGYKCDSSGNMKYLVYGIPGDKTRKDQPFKGKSGFVTWIESKENNLGYWLMFYDYKTNNILIPVK

**Protein ID 31**: Putative uncharacterised protein *C. sporogenes* PA 3679

MFNYYNPCSMCMPIPIYLK**GPHPMAPMGYGYDHVSETK**ENDDDLNCLYPKIYFKVYPLVKRHCDMMEREKGKDYCPCEKEVDEACKEIYKRIKPELDEDEDDYSRQRRYRRRHAVNDLIRIILINELFGRRRRRRRRRRRPYDYNPNYNYNYSYDNYDDYDDYYDFDDDDD

**Protein ID 45**: Exosporium protein BclB *C. sporogenes* PA 3679

MSHRCK**MICMPCCCNCTCPR**GVTGPTGPRGITGPTGPIGITGPTGPIGITGPTGPTGVTGPTGPIGITGTTGPIGITGPTGPTGASAIIPFASGGPVALVTVLGGLANTGALLGFGSSFPGVTVSAGTITLSPTVSDFAFVAPRTGTITSLAGFFSATVGVTLLSPVQIRLTIYTAPAASNTFTPVGTPLLLTPALGVIAIGTTASGITAEAIPVAAGDKILLVADSDTLGVSLASTVTGYVSAGIAIS

**Protein ID 50:** Uncharacterized protein *C. sporogenes* ATCC 15579

MLCFGVIKMSR**INIFTGHFGSGK**TEIAINYAMKLAKEGKKVALVDIDIVNPYFCSRSLKEEFDKLGIRVIASDSKLMNAELMVVPGEVMAVFNDKSYEVVMDIGGDDQGATVLGQYNKYFNEEDYDMYFVVNNNRPLTSNEKETEDYIKSIEISSRLKVKYLISNTNLSYETTVDHILKGDEIVLELSKKTGLPYKYIVCRKDFLDDIKGKVHGEIFPIDIYMKPPWR

**Protein ID 51:** uncharacterised protein (4 cysteine residues)

N Unused Total Uniprot Accession #

51 2.00 2.00 J7T3Q4_CLOSG Uncharacterized protein OS=Clostridium sporogenes ATCC 15579

51 0.00 2.00 J7T3K8_CLOSG Uncharacterized protein OS=Clostridium sporogenes ATCC 15579

51 0.00 2.00 J7SGB6_CLOSG Uncharacterized protein OS=Clostridium sporogenes ATCC 15579

51 0.00 2.00 G9F472_CLOSG Putative uncharacterized protein OS=Clostridium sporogenes PA 3679

51 0.00 2.00 G9F2S4_CLOSG Putative uncharacterized protein OS=Clostridium sporogenes PA 3679

51 0.00 2.00 G9EZR9_CLOSG Putative uncharacterized protein OS=Clostridium sporogenes PA 3679

J7T3Q4_CLOSG

MADKNITCKDCGK**EFVFTEGEQEFYK**EKGFENEPQRCPDCRRARKQARNNNRSFR

J7T3K8_CLOSG

MADKTLTCKDCGK**EFVFTEGEQEFYK**EKGFENEPQRCPECRRARKQERNNNRGFRR

J7SGB6_CLOSG

MADKNITCKDCGK**EFVFTEGEQEFYK**EKGFENEPQRCPECRKARKQQNNRGFRR

G9F472_CLOSG

MADKNITCKDCGK**EFVFTEGEQEFYK**EKGFENEPQRCPECRKARKQQNNRGFRR

G9F2S4_CLOSG

MADKNITCKDCGK**EFVFTEGEQEFYK**EKGFENEPQRCPDCRRARKQARNNNRSFR

G9EZR9_CLOSG

MADKTLTCKDCGK**EFVFTEGEQEFYK**EKGFENEPQRCPECRRARKQERNNNRGFRR

**Protein ID 55**: Uncharacterized protein *C. sporogenes* PA 3679

MSKRCRCKSKSRCRGRKGSGNGLIILILILLQFGCCR**GFHNECHGGR**RGCCEVDNSILFIIALYYLSCCGDFSLCC

**SI-3. Proteins identified from unwashed exosporium**

Unwashed exosporium fragments were loaded on to SDS gel (supplementary Fig. 1) and 6 major bands were analysed by MALDI-TOF analysis. The high score protein IDs were shown below. The Amino acid sequences shown in bold are peptides identified.

**Gel band ID-1, Accession number gi|187778200 hypothetical protein CLOSPO_01792 [*C. sporogenes* ATCC 15579], molecular weight 59.3 kDa**

M**ANSIVFSNSAEELKTSVFGYDGSNYLPLK**VNQYGELDINVGTISKIDSITSGTVNVTQVNSLTNGTITKVSSITNGTVDVTQLNSLTNGTITKVSSITNGTVDVTQLSSLTNGTIAKVSSITNGTVDVTQLSSLTSGTVNVTQVNSLTNGTITKVSSITNGTVDVTQLSSLTNGTITKVSSITNGTVDVTQLSSLTSGTIAKVSSITNGTVDVTQLSSLTNGTITKVSSITNGTVDVTQLSSLTSGTIAKVDSITNGTVDVTQLSSLTNGTITKVSSITNGTVDVTQLSSLTNGTVDVTQLSSLTNGTIAKVSSITNGTVDVTQLSSLTNGTITKVSSITNGTVDVTQLSSLTNGTITKVSSITNGTIDVTQLSSLTSGTIAKVDSITNGTVDVTQLSSLTNGTITKVSSITNGTVDVTQLSSLTSGTIAKVDSITNGTVDVTQLNSLTSGTIAKISSITGGSINVNITDRYFNETSETVSTIALATPQYSTAVDTSK**MQDITWYVK**KVAGGTMPVTVSIAVCPEQNGTYVLVGETATVGAVGTAKALSSSYYMHYTKVYCTTAATTNQSVQVFFNGR

**Gel band ID-2, Accession number i|187779073 hypothetical protein CLOSPO_02668 (Peptidase M24)[*C. sporogenes* ATCC 15579], molecular weight 67.8 kDa**

MKVSERLTKLRTLMTEKNIDMYIVPTADFHQSEYVGEHFKAR**KYITGFSGSAGTAVITKDHAGLWTDGR**YFLQAGNQLKGTTVELFKMGEPGVPTIEEYIMNTLPDKGTLGFDGRVVSMGDGQTYEKILSSKNADINYDCDLINDIWEDRPPLSEEPAFELDIKYTGESTASKLKRIREAMTAEGANTHVITSLDDIAWTLNIRGNDIEFFPLILSYLIITMDEVHLFINETKLSDEIK**SNLKENGVSFIHPYNEVYETVK**KFTNSDVVLVDPARMNYALYNNIPEDVKKVEKR**NPSVLFK**AMK**NPIEIENIKK**AQIKDGVAHTKFMYWLKHNIGK**EVITEISASNKLDEFR**AEQGGFIRPSFEPISSFGEHAAIVHYAPTPETDIELKEGSLFLTDTGAGFYEGSTDITRTYALGEVPQIMKDHFTLTVNSNLHLAHARFLYGCNGMNLDILAR**APFWNR**ALNFNHGTGHGVGYLMNIHEAPTGFRWQYRANETHPFEEGMVITDEPGIYIAGSHGVRIENELLVCKGEKNEYGQFMYF EPISYVPIDLDAINPDLMTTEEKTWLNEYH EIVYNTISPYLTQEEKDWLKEYTKKIS

**Gel band ID-3, Accession number gi|148381241 chaperonin GroEL [*C. botulinum* A str. ATCC 3502], molecular weight 57.9 kDa**

MAKSLLFGEQARRSMEAGVDKLADTVRVTLGPKGRNVVLDKK**FGSPLITNDGVTIAR**EIELEDPYENMGAQLVKEVATK**TNDVAGDGTTTATLLAQAIIREGLKNVTAGANPIQIR**TGIRKAVEKAVEEIKVISKPVNGKEDIAR**VAAISAASEEVGK**LIADAMER**VGNDGVITVEESK**SMGTDLEVVEGMQFDRGYVSAYMVTDTEKMEAVLDDVYILITDKKISNIQEILPILEQIVQQGKKLLIISEDIEGEALSTLVLNKLRGTFTCVGVKAPGFGDRRKEMLQDIAILTGGEVISEELGRDLKDVTIDMLGTADSVKVTKENTTIVNGKGDKVAIKERVSQIRVQIEDTTSEFDKEKLQERLAK**LAGGVAVIRVGAATETELKEEKLRIEDALAATKAAVEEGIVPGGGTAYIDIIPK**IADLTSDIIDVK**LGIDIIRKALEEPVR**QIANNAGAEGSVIIEKVKATEAGVGYDALNDKYVDMLKTGIVDPTKVTRSALQNAASIASTFLTTEAAVADIPEKENTPPMAPGMGMDGMY

**Gel band ID-4, Accession number gi|148379770 glutamate dehydrogenase, NAD-specific [*C. botulinum* A str. ATCC 3502], molecular weight 45.5 kDa**

M**AKENLNPFENAQK**QVKTACDKLGMEPAVYELLKEPQR**VIEVSIPVK**MDDGSVKVFKGYRSQHNDAVGPTK**GGVRFHPNVSLDEVK**ALSIWMTFKCSVTGIPYGGGK**GGIIVDPKTLSKGELER**LSR**GYIDGIHK**LIGEKVDVPAPDVNTNGQIMAWMVDEYNKLVGR**SAIGVITGKPVEFGGSLGRNAATGFGVAVTAR**EAAAKLGIDMKKAKLAIQGIGNVGSHTVLNCEKLGGTVVALAEWCKEEGTYAIYNENGLDGKAMIEYVKENGNLLGYPGAK**KISLDEFWALNVDILIPAALENAITHENASSINAK**LVCEAANGPITPDADAILKEKGITVTPDILTNAGGVTVSYFEWVQNLYGYYWTEAEVEAKEEEAMVK**AFESIWAIK**EEYSVTMREAAYMHSIKKVAGAMK**LRGWY**

**Gel band ID-5, Accession number gi|187778820 hypothetical protein CLOSPO_02415 (Ornithine carbamoyltransferase)[*C.* *sporogenes* ATCC 15579],**

MDFTPK**EINYFLDLAR**DLKRAKYTGTEVQRLKGK**NIALIFEK**ASTRTRCAFEVGAK**DQGAHVTYLGPTGSHIGK**KESAADTARVLGR**MYDGIEYR**GFGQEIVETLAEYAGVPVWNGLTDEDHPTQILADFLTIR**EHFNKPLSEIK**FAYVGDGANNMANALMIGAVKMGMDFRIVSPK**EIPTDAALVAK**CKEIAAETGAK**VTITDNIEEGVK**GCDVLYTDVWVSMGEPDSVWESKIKLLTPYRVDMNMIKMTGNPDAKFMHCLPAFHDEETAVGKEIKEKYGLSEMEVSHELFESK**YSIVFDEAENR**MHTIKAVMVATLGDQ

**Gel band ID-6, Accession number gi|148380430** **proline racemase [C. *botulinum* A str. ATCC 3502], molecular weight 36.4**

MRAIKTIQTIESHTMGEPTRIVIGGLPKVPGKTMAEKMEYLEENNDSLRTMLMSEPRGHNDMFGAIYTEPADETADLGIIFMDGGGYLNMCGHGSIGAATCAVEMGIVK**VEEPYTNIK**LEAPAGMINARVKVEDGKAKETSIVNVPAFLYKK**DVEIDVPDYGK**LTLDISFGGSFFAMVDAEK**VGIDISPANSQK**LNDLGMK**IVHAVNEQVEIK**HPVLEHIKTVDLCEFYGPAKSEDADVQNVVVFGQGQVDRSPCGTGTSAKMALLYAQGKMKVGEEIVNESIICTKFKGKILEETKVGEYDGIIPEITGSAYVTGFSQFLVDEEDPVKYGFVLK

**Reference:**

1. The UniProt Consortium., 2011. Ongoing and future developments at the Universal Protein Resource. Nucleic Acids Res. 39, 214-219.
