# Supplementary material for: Characterization of the spore surface and exosporium proteins of Clostridium sporogenes; implications for Clostridium botulinum group I strains
Source: Food Microbiol. 2016 Oct;59:205–12. doi: 10.1016/j.fm.2016.06.003 (PMC4942563; doi:10.1016/j.fm.2016.06.003)
Supplement: Supplementary file 2 [file mmc2.docx]

**Supplementary Figures**

**Fig S1. Proteins of unwashed exosporium**

French pressed, unwashed exosporium samples were treated with 8 M urea, 4 M DTT and 10% SDS for 20 min at 95°C, and proteins separated by SDS PAGE (4-12%; NuPAGE-Invitrogen) with MOPS running buffer. The gel was stained with Coomassie brilliant blue R-250. Lane MW contained molecular weight markers, as indicated on the left. Lane 1 contained exosporium proteins; bands 1-6, corresponding to IDs 1-6, were excised and analysed by LC-MS/MS (Table 1 of main text). Protein sequences and identified peptides are shown in Supplementary Information 3.

**
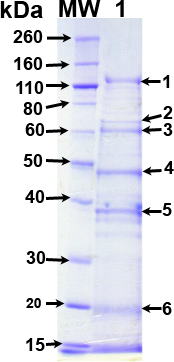
**

**Fig S2. Multiple sequence alignment (Clustal O (1.2.1) of BclB proteins.**

BclB, a collagen-like triple-helix-repeat-containing protein from *C. sporogenes* PA 3679 (EHN3755.1) is aligned with its homologue in *C. botulinum* Prevot 594 (AJD29680.1) and with BclB of *B. anthracis* (WP_047957069.1). The C-terminal domain is strongly conserved. Alignment constructed with Clustal Omega (http://www.ebi.ac.uk/Tools/msa/clustalo/).

WP_047957069.1 MKHNDCFGHNNCNNPIVFTPDCCNNPQTVPITSEQLGRLITLLNSLIAAIAAFFANPSDA

EHN13755.1 ------------------------------------------------------------

AJD29680.1 ------------------------------------------------------------

WP_047957069.1 NRLALLNLFTQLLNLLNELAPSPEGNFLKQLIQSIINLLQSPNPNLGQLLSLLQQFYSAL

EHN13755.1 ------------------------------------------------------------

AJD29680.1 ------------------------------------------------------------

WP_047957069.1 APFFFSLILDPASLQLLLNLLAQLIGVTPGGGATGPTGPTGPGGGATGPTGPTGPTGPGG

EHN13755.1 -----------MSHRCKMICMPCCCNCTCPRGVTGPTGPRG----ITGPTGPIGITGP--

AJD29680.1 -----------MSHRCKMICMPCCCNCTCPRGVTGPTGPRG----ITGPTGPTGVTGP--

* : : : * *.****** * ****** * ***

WP_047957069.1 GATGPTGATGPTGDTGLAGATGATGPTGDTGVAGPAGPTG---PTGDTGLAGATGPTGPT

EHN13755.1 --TGPIGITG--------------------------------------------------

AJD29680.1 --TGPIGITGPTGPIG------ITGPTGPIGITGPTGPIGITGPTGPIGITGPTGPIGIT

*** * **

WP_047957069.1 GDTGLAGATGPTGATGLAGATGPTGATGLTGATGATGAAGGGAIIPFASGTTPAA-LVNA

EHN13755.1 -PTGPTGVTGPTGPIGITG------TTGPIGITGPTGPTGASAIIPFASGGPVALVTVLG

AJD29680.1 GPTGPIGITGPTGPIGVTG------PTGPIGITGPTGPTGASAIIPFASGGPVALVTVLG

** * ***** *::* ** * ** ** :*..******** * * .

WP_047957069.1 LIANTGTLLGFGFSQPGIALTGGTSITLALGVGDYAFVAPRDGVITSLAGFFSATAALAP

EHN13755.1 GLANTGALLGFGSSFPGVTVSAGT-ITLSPTVSDFAFVAPRTGTITSLAGFFSATVGVTL

AJD29680.1 GLANTGALLGFGSSFPGVTVSAGT-ITLSPTVSDFAFVAPRTGTITSLAGFFSATIGVTL

:****:***** * **::::.** ***: *.*:****** *.*********** .::

WP_047957069.1 LSPVQVQIQILTAPAASNTFTVQGAPLLLTPAFALIAIGSTASGIIPEAIPVAAGDKILL

EHN13755.1 LSPVQIRLTIYTAPAASNTFTPVGTPLLLTPALGVIAIGTTASGITAEAIPVAAGDKILL

AJD29680.1 LSPVQIRLTIYTAPAASNTFTPVGTPLLLTPALGIIAIGTTASGITAENIPVAAGDKILL

*****::: * ********** *:*******:.:****:***** * ***********

WP_047957069.1 YVSLTA--ASPIAAVAGFVSAGINIV

EHN13755.1 VADSDTLGVSLASTVTGYVSAGIAIS

AJD29680.1 VADSDTLGVDLASVVTGYVSAGIAIS

.. : .. :.*:*:***** *

An * (asterisk) indicates positions which have a single, fully conserved residue.

A : (colon) indicates conservation between groups of strongly similar properties

A . (period) indicates conservation between groups of weakly similar properties

**Fig S3. Sequence alignment (Clustal O (1.2.1) of CsxA proteins**. The newly-described CsxA of C. *sporogenes* NCIMB 701792 is aligned with *C. botulinum* homologues. CsxA has 84 % identity to gi|152935245 of *C*. *botulinum* F Str. Langeland, 100% identity to gi|745830402 of *C. botulinum* Prevot 594, 87% identity to gi|757418180 of *C. botulinum* B2 450 and 86% identity to gi|559782037 of *C. botulinum* B Str. Osaka05.

gi|152935245 MAINSKDFIPRPGFVNKQGCLPDPVEITCIQVPKVFDQCLIKECLKPTDDCEQLCKQIPN

701792-CsxA MAINSKDFIPRPGFVNKQGCLPDPVEICCIQVPKVFDQCLRKECLKPTDDCEQLCKQIPN

gi|745830402 MAINSKDFIPRPGFVNKQGCLPDPVEICCIQVPKVFDQCLRKECLKPTDDCEQLCKQIPN

gi|757418180 MAINSKDFIPRPGFVNKQGCLPDPVEITCIQVPKVFDQCLIKECLKPTDDCEQLCKQIPN

gi|559782037 MAINSKDFIPRPGFVNKQGCLPDPVEITCIQVPKVFDQCLIKECLKPTDDCEQLCKQIPN

*************************** ************ *******************

gi|152935245 VKEPAQVRSVGCCKDLKVKINSVTKCPVSNGKPGHKKITINFTVTFDVDVEVEKHGVKRT

701792-CsxA ITDPSQVRCVGCCKNLKVIVNSVTKCPVSNGKPGYKKVTINYTITFDVDVDVEINGVTQT

gi|745830402 ITDPSQVRCVGCCKNLKVIVNSVTKCPVSNGKPGYKKVTINYTITFDVDVDVEINGVTQT

gi|757418180 ITDPAQVRCVGCCKDLKVKVNSVTKCPVSNGKPGHKKVTINFTVTFDVDVDVEINGVIHT

gi|559782037 ITDPAQVRCVGCCKDLKVKVNSVTKCPVSNGKPGHKKVTINFTVTFDVDVDVEINGVIHT

:.:*:***.*****:*** :**************:**:***:*:******:** .** :*

gi|152935245 ETLKYSVNRTITAPNLYCPDAIAKTIIGKECTSAEEVDQQFIKLEVVGECLSTDITKVDC

701792-CsxA QTLSYSVNRTITASNLYCPDTIAKTIIGKECTSAEEVDQQFIKIEVVGDCLSTDISKIDC

gi|745830402 QTLSYSVNRTITASNLYCPDTIAKTIIGKECTSAEEVDQQFIKIEVVGDCLSTDISKIDC

gi|757418180 ETLNFSVNRTITASNLYCPDAIAKTIIGKECTSAEEIDQQFIKLEVVGECLSTDISKIDC

gi|559782037 ETLNFSVNRTITASNLYCPDTIAKTIIGKECTSAEEIDQQFIKLEVVGECLSTDISKIDC

:**.:******** ******:***************:******:****:******:*:**

gi|152935245 DKDCCSCTCTCEDD---EDKKVFLCITLGLFIIIKCEIVVQLMVPTYGYCPVPEECKRSH

701792-CsxA GGGC-NCGCTCPDDPNNGDNKVFLCITLGLFIIIKCEIVVQLMVPAYGYCPVPEECKCSH

gi|745830402 GGGC-NCGCTCPDDPNNGDNKVFLCITLGLFIIIKCEIVVQLMVPAYGYCPVPEECKCSH

gi|757418180 DNDCCSCSCTCE---DNGDKKVFLCITLGLFIIIKCEIVVQLMVPAYGYCPVPEECKCSH

gi|559782037 DNDCCSCSCTCE---DTGDKKVFLCITLGLFIIIKCEIVVQLMVPAYGYCPVPEECKCSH

* .* *** *:*************************:*********** **

gi|152935245 DPCKEFMERELPTLYPPQEMDNLFDDCDEKCIEE-----EE-EDDIVSDSIVSSSSIISSN

701792-CsxA DPCKEFMERELPTLYPPQEMDNLFDEYDERQDDRHIHNEKHIEEEEERGNMITSS VITNN

gi|745830402 DPCKEFMERELPTLYPPQEMDNLFDEYDERQDDRHIHNEKHIEEEEERGNMITSS-VITNN

gi|757418180 DPCKEFMERELPTLYPPQEMDNLFDDYDERQDERHIHDRKHIEEEEERGNLVTSS-IIASN

gi|559782037 DPCKEFMERELPALYPPQEMDNLFDDYDERQDERHIHDREHIEE-EERGNLVTSS-IITSN

************:************: **: :. *: . ::::** :*:.*

An * (asterisk) indicates positions which have a single, fully conserved residue.

A : (colon) indicates conservation between groups of strongly similar properties

A . (period) indicates conservation between groups of weakly similar properties
